# Stepwise double excited-state proton transfer is not possible in 7-azaindole dimer†
†Electronic supplementary information (ESI) available: Survey of experimental data; molecular structures; spectroscopic characterization; dynamics analysis. See DOI: 10.1039/c5sc01902h


**DOI:** 10.1039/c5sc01902h

**Published:** 2015-07-08

**Authors:** Rachel Crespo-Otero, Nawee Kungwan, Mario Barbatti

**Affiliations:** a School of Biological and Chemical Sciences , Queen Mary University of London , Mile End Road , London E1 4NS , UK . Email: r.crespo-otero@qmul.ac.uk; b Department of Chemistry , Faculty of Science , Chiang Mai University , Chiang Mai 50200 , Thailand . Email: naweekung@hotmail.com; c Max-Planck-Institut für Kohlenforschung , Kaiser-Wilhelm-Platz 1 , D-45470 , Mülheim an der Ruhr , Germany . Email: barbatti@kofo.mpg.de

## Abstract

The nature of the excited-state double proton transfer in 7-azaindole (7AI) dimer—whether it is stepwise or concerted—has been under a fierce debate for two decades. Based on high-level computational simulations of static and dynamic properties, we show that much of the earlier discussions was induced by inappropriate theoretical modelling, which led to biased conclusions towards one or other mechanism. A proper topographical description of the excited-state potential energy surface of 7AI dimer in the gas phase clearly reveals that the stepwise mechanism is not accessible due to kinetic and thermodynamic reasons. Single proton transfer can occur, but when it does, an energy barrier blocks the transfer of the second proton and the dimer relaxes through internal conversion. Double proton transfer takes place exclusively by an asynchronous concerted mechanism. This case-study illustrates how computational simulations may lead to unphysical interpretation of experimental results.

## Abstract 2

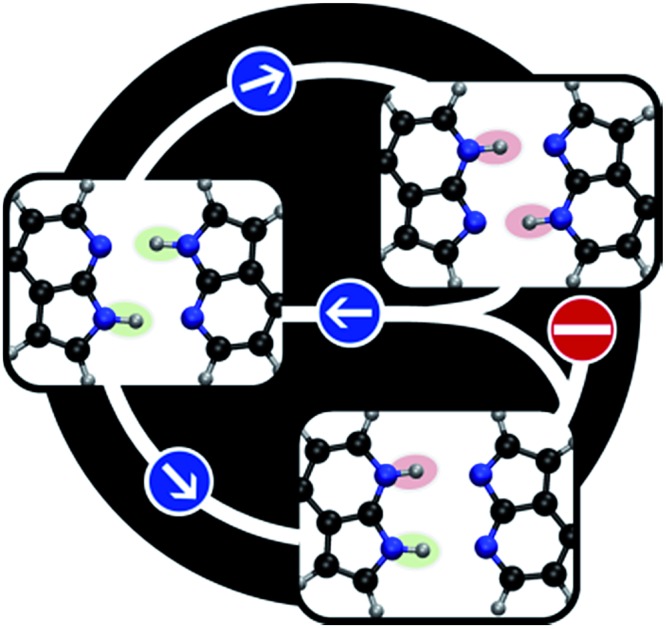
Topographical analysis of the dimer's excited state shows that internal conversion after first proton transfer blocks the stepwise process.

## 1. Introduction

**Fig. 1:**
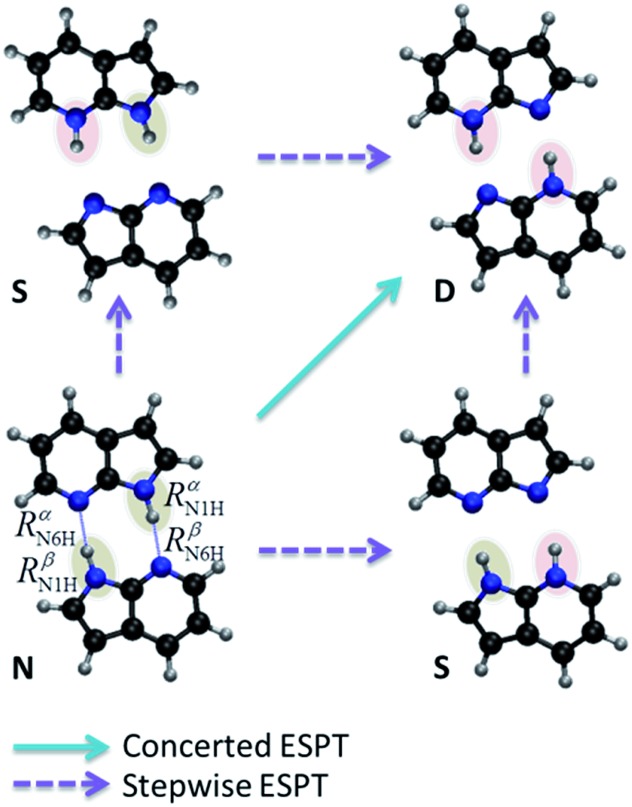
Schematic double proton transfer in 7AI dimer. The transfer may occur *via* a concerted or stepwise mechanism.

A central problem in physical chemistry is to understand how photoinduced multiple proton transfers take place in dimers. For decades,^1^–^4^ 7-azaindole (7AI) dimer has been adopted by experimentalists and theorists as a prototype for investigating such processes. After Zewail and co-workers,^2^ based on time-resolved spectroscopy and computational modelling, proposed that photoexcitation near the band origin induces a stepwise double proton transfer in 7AI dimer in the gas phase (Fig. 1), a heated debate took place between advocates of concerted^5^ and stepwise mechanisms.^6^ This debate, however, has been shifted to the excited-state proton transfer (ESPT) of the 7AI dimer in condensed phases,^4^,^6^,^7^ even though a consensus has never been settled as to the nature of the proton transfer in the gas phase.

Experimental results show that the 7AI dimer in the gas phase excited near the band origin has an ultrafast dynamics with short (0.2–0.6 ps) and long (1–3 ps) time components^2^,^8^–^10^ (a survey of time-resolved experimental data is given in Section S1 of the ESI†). The interpretation of these results has been under dispute for two decades.^11^ Part of the problem is that, so far, *all theoretical models guiding the experimental analysis failed to provide a balanced description of the several different diabatic regions of the first excited state*. As we show below, this imbalance led to prediction of spurious minima, missing conical intersections, wrong descriptions of charge-transfer structures; all of that contributing to biased discussions of the mechanisms.

Based on state-of-the-art quantum-chemical simulations, we readdress the ESPT of 7AI dimer in the gas phase. We show that the stepwise mechanism is not accessible in the excited state due to kinetic and thermodynamic reasons. Single proton transfer can occur, but when it does, an energy barrier blocks the transfer of the second proton and the dimer relaxes through internal conversion. As a result, double proton transfer can only take place through concerted mechanisms.

## 2. Methods

Excited states were computed with the coupled cluster to approximated second order (CC2)^12^ and with the algebraic diagrammatic construction to the second order [ADC(2)],^13^,^14^ both using the resolution-of-the-identity (RI) approximation.^15^ In the case of the ADC(2), the corresponding ground state was computed at the second-order Møller–Plesset perturbation (MP2) theory.^12^ CC2 calculations were done with the TZVP basis set.^16^ ADC(2) calculations were done with the SV(P) and TZVP basis sets. Conical intersections were optimized with the penalty Lagrange multiplier technique (*α* = 0.02 Hartree) implemented in the CIOPT program,^17^ which we have adapted to work with CC2 and ADC(2). The impact of the main approximations employed in CC2 and ADC(2) were evaluated by computing the *D*_1_,^18^*D*_2_,^19^ and %*τ*_2_(ref. 20) diagnostics.

Exploratory dynamics simulations in the excited states were also computed. First, the absorption spectrum was simulated at the ADC(2)/SV(P) level with the nuclear ensemble method^21^ (ESI, Section S2†). Initial conditions were sampled from two energy windows in the spectrum: 4.1 ± 0.1 eV (A) and 4.7 ± 0.1 eV (B). The initial states were determined according to the distribution of oscillator strengths within each window. In window (A), 20 trajectories were initiated in S_1_. In window (B), 7 trajectories were initiated in S_2_, 12 in S_3_ and 5 in S_4_, in a total of 24 trajectories. Due to the reduced number of trajectories, all dynamics results have low statistical significance and they should be understood as a qualitative exploration of the potential energy surfaces. This qualitative aspect, however, does not undermine our main conclusions, which are based on the analysis of high-level potential energy surfaces.

On-the-fly dynamic simulations were carried out in the excited states computed with the ADC(2)/SV(P) level of theory.^22^,^23^ Starting in window (A), only the S_1_ state was considered. Starting in window (B), all excited states up to S_4_ were included. Nonadiabatic effects were taken into account by the surface hopping approach. Classical equations were integrated with 0.5 fs time step, while quantum equations were integrated with 0.025 fs using interpolated quantities between classical steps. The maximum simulation time was 1000 fs. Hopping probabilities were computed with the fewest switches approach^24^ including decoherence corrections.^25^ Nonadiabatic couplings with ADC(2) were computed with the method discussed in ref. 22 based on the Hammes-Schiffer/Tully approach.^26^

CC2 and ADC(2) calculations were carried out with the TURBOMOLE program.^27^ The spectrum and dynamics simulations were performed with NEWTON-X^28^,^29^ interfaced with TURBOMOLE. Further details on the computational methods are given in the ESI, Section S2.†

## 3. Results

### 3.1 The excited-state potential energy surface

**Fig. 2:**
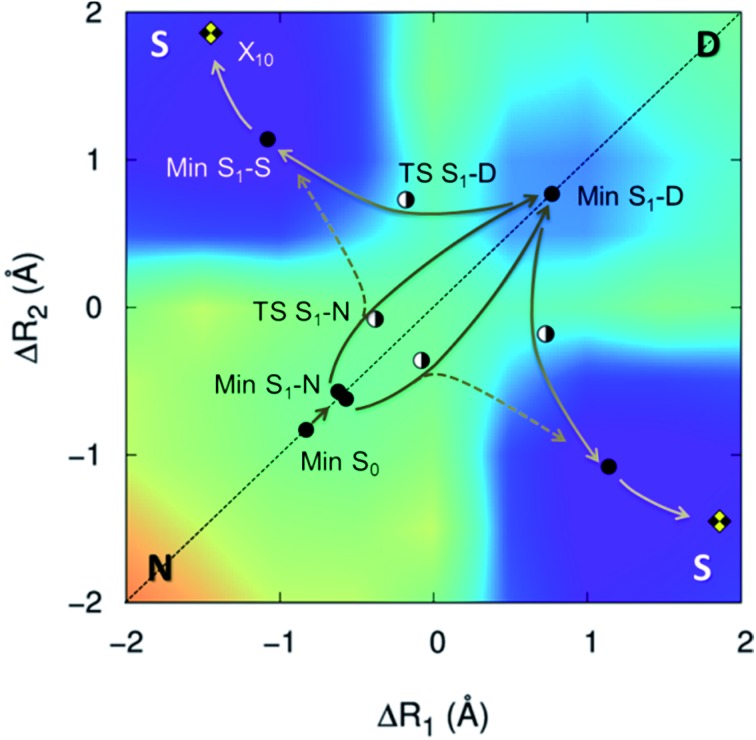
S_1_ potential energy surface of 7AI dimer. Stationary structures and conical intersections with the ground state are indicated by points. The energy grows from violet/blue to yellow/orange.

The proton transfers in 7AI dimer can be conveniently discussed in terms of the Δ*R*_1_ – Δ*R*_2_ plane defined by the internal coordinates Δ*R*_1_ = *R*αN1H – *R*βN6H and Δ*R*_2_ = *R*βN1H – *R*αN6H (Fig. 1), where *R*mN1H is the NH distance in the pyrrole group of monomer m and *R*nN6H is the NH distance in the pyridine group of monomer n. The main tautomers of 7AI dimer—the normal dimer (**N**), the single proton transfer (**S**), and the double proton transfer (**D**)—lie in separated regions of the Δ*R*_1_ – Δ*R*_2_ plane (Fig. 2), facilitating the analysis.

**Fig. 3:**
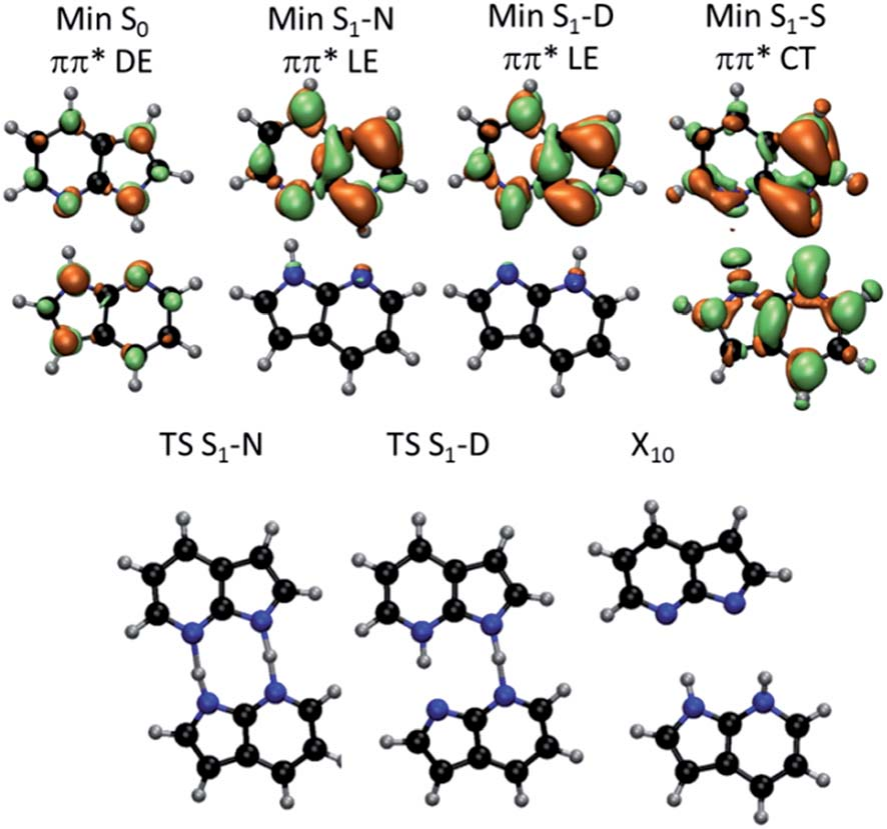
(Top) S_1_–S_0_ electronic density difference at the S_1_ minima of 7AI dimer. Orange regions are electron donor. Green regions are electron acceptor. (Bottom) Geometries of the S_1_ transition states and S_1_/S_0_ conical intersection. DE – delocalized excitation; LE – localized excitation; CT – charge transfer.

Excited-state geometries were optimized with CC2/TZVP (ESI, Section S3†) and energies were computed at the same level (Table 1 and ESI, Sections S4 and S5†). In the Δ*R*_1_ – Δ*R*_2_ plane, the S_0_ minimum lies on the diagonal line (Fig. 2) and the S_1_ state is a delocalized π–π* state belonging to the B_u_ representation of the *C*_2h_ point group (Fig. 3). The allowed B_u_ vertical transition lies at 4.577 eV and the B_u_–A_g_ exciton splitting is only 0.02 eV, as also obtained with multi-reference perturbation theory (MRMP).^30^

**Table 1:** Potential energies at the minima, transition states, and crossing geometries on S_1_ computed with CC2/TZVP

Geometry	S_0_ (eV)	S_1_ (eV)	Δ*E* (eV)	*f*
Min S_0_	0.000	4.577	4.58	0.100
Min S_1_-N	0.520	4.142	3.62	0.062
Min S_1_-S	1.938	3.216	1.28	0.002
Min S_1_-D	1.177	3.602	2.43	0.015
X_10_	3.367	3.404	0.04	—
TS S_1_-N	0.895	4.191	3.30	—
TS S_1_-D	1.235	4.139	2.90	—

From the S_0_ minimum, a S_1_ minimum before proton transfer (Min S_1_-N) can be reached by a small relaxation with symmetry breaking. Due to this symmetry breaking, the π–π* state localizes over one monomer, as experimentally observed by Sakota and Sekiya.^31^ The adiabatic excitation into this minimum is 4.142 eV (Table 1), in good agreement with the experimental band origin assigned at 3.999 eV (32 252 cm^–1^).^32^ Another S_1_ minimum with similar character lies along the diagonal line, corresponding to the double-proton-transferred (PT) tautomer (Min S_1_-D). The adiabatic excitation for this minimum computed from the S_0_ minimum of the **D** structure is 3.048 eV at CC2/TZVP (not shown in Table 1), in fair comparison to the experimental assignment at 2.860 eV (23 071 cm^–1^).^32^ The single-PT structure (**S**) has also a corresponding S_1_ minimum, but with strong charge-transfer (CT) character (Min S_1_-S, Fig. 3).

From the Min S_1_-N, a transition state (TS S_1_-N, Fig. 3) can be reached. It lies close to the diagonal line and should preferentially lead to the double-PT structure **D**, although it may also be a gate to **S** structures. A second transition state lies between the S_1_ minima **D** and **S** (TS S_1_-D).

The CT character of the single-PT structure (**S**) should allow for the Sobolewski–Domcke proton-coupled electron transfer internal conversion mechanism.^33^ In fact, a search for conical intersections in the **S** region reveals that the seam between S_1_ and S_0_ (X_10_, Fig. 3) lies nearby, only 0.2 eV higher than Min S_1_-S.

The potential energy surface shown in Fig. 2 was obtained by fixing the Δ*R*_1_ and Δ*R*_2_ coordinates and optimizing all others at the CC2/TZVP level (ESI, Section S6†). One of the most significant features of this surface is that there is no high-energy S_1_ minimum for single transfer. The existence of such a minimum would be fundamental for occurrence of a stepwise mechanism.

### 3.2 Ballistic excited-state proton transfer

**Fig. 4:**
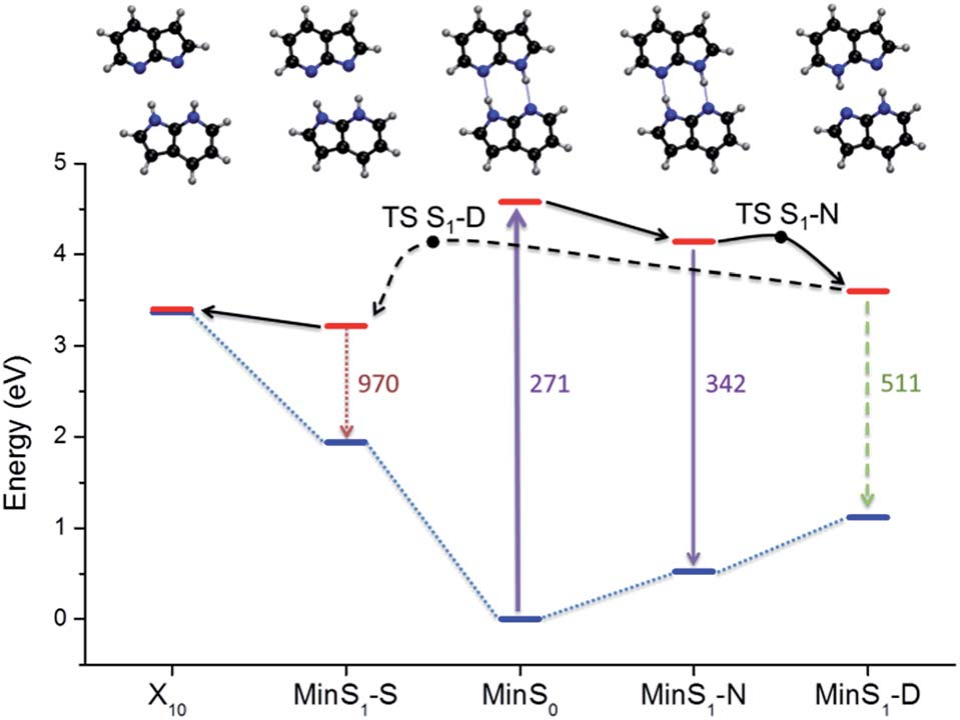
Potential energy diagram including the main stationary points and conical intersections.

The relative energy of the stationary points and conical intersections on the S_1_ surface indicates that the **N** structure is separated from **D** by a 0.05 eV barrier only (Fig. 4), making the concerted path easily available. The **S** structure is more stable than **D** by 0.4 eV, creating a clear thermodynamic trend from **D** to **S**, rather than the opposite as supposed by the stepwise hypothesis. For **S** to convert into **D**, a 0.9 eV barrier should be overcome. The fate of the **S** structures, therefore, should be internal conversion at X_10_.

To verify these predictions including kinetic effects, we have run surface hopping dynamics in the above-the-barrier limit, where ballistic PT is more relevant than tunneling PT.^34^ To cope with the high computational cost, dynamics was done at ADC(2)/SV(P) level. This level reproduces all stationary points and conical intersections predicted by CC2, with a 0.1 eV energy overestimation. Two windows of initial energy were investigated, 4.1 ± 0.1 eV (A) and 4.7 ± 0.1 eV (B).

As expected from the analysis of the S_1_ topography, the concerted mechanism dominates the dynamics, as the time lag between the first and the second PT is always smaller than 20 fs (Table 2). This short but non-zero time lag implies that (1) the concerted transfer is asynchronous;^4^ (2) there is no time for formation of a stable intermediate; (3) the second transfer is strongly correlated^4^ to the first. *Notice yet that the occurrence of concerted PT in the ballistic regime does not disprove the stepwise mechanism in the tunneling regime*.^34^ The arguments against the stepwise mechanism are given in the next section.

**Table 2:** Dynamics results in the two excitation windows. SPT, DPT and MPT indicate single, double, and multiple proton transfers. *τ*_PT1_ and *τ*_PT2_ are the average times for the first and second PT in each class

Window A	SPT	DPT	MPT
*τ*_PT1_ (fs)	78	90	45
*τ*_PT2_ (fs)	—	101	49
Yield (%)	15	80	5

Window B	SPT	DPT	MPT
*τ*_PT1_ (fs)	50	125	115
*τ*_PT2_ (fs)	—	141	135
Yield (%)	8	54	38

All trajectories exhibiting at least two proton transfers (85% and 92% in windows (A) and (B), respectively) featured a concerted mechanism. A minor fraction of the trajectories in both windows (15% and 8%) underwent a single PT and formed an **S** structure from **N**. **S** structures were also indirectly formed from **D** in 5% of the trajectories in the low-energy window (A). This fraction grew to 38% in the high-energy window (B), reflecting the role of the energy barrier separating **D** from **S**. Examples of trajectories are discussed in the ESI, Section S7.†

Whatever the source of **S** is, all those structures tend to undergo internal conversion to the ground state within 1 ps. They reached the X_10_ intersection in average 140 fs after forming the single-PT structure (ESI, Sections S8 and S9†). The population flow from **D** to **S** followed by internal conversion in **S** explains why the fluorescence quantum yield of the **D** tautomer is reduced by a factor 10 in comparison to that of the 7AI monomer.^35^

## 4. Discussion

### 4.1 Proton-transfer mechanisms in 7AI dimer

The topography of the excited state of 7AI dimer shows that there are four reasons why excited-state stepwise PT is not possible in the gas phase:

(1) The **S** structure is more stable than the **D** structure in the S_1_ state, creating a thermodynamic trend which blocks the second PT in the stepwise process.

(2) There is no high-energy **S** local minimum in the S_1_ state, which could work as an intermediate for the stepwise mechanism.

(3) A low-energy intersection seam with the ground state lies in the **S** region, implying that internal conversion should be the fate for the **S** structures.

(4) Starting from **N**, the transition state on the S_1_ state is displaced towards **D**, creating a kinetic bias towards concerted paths.

This topography is still compatible with a fraction of the population undergoing single-PT tunneling, as proposed in ref. 2. Nevertheless, since the formed **S** structures relax through internal conversion, they cannot be the source of double-PT structures **D**. Thus, any **D** structure should exclusively arise from concerted double PT of the remaining population.

Based on these results, we have developed the following hypothesis for the origin of the two experimentally observed time constants: (1) the short time constant (0.2–0.6 ps) should be related to ballistic (or maybe near-edge tunneling) concerted **N** → **D**, using the energy excess of the low-energy-resolved fs-laser pulses, as suggested by Sekiya and coworkers;^10^,^11^ (2) the long time constant (1–3 ps) should be related to tunneling rate at deeper levels, probably composed of two contributions, **N** → **S** (as proposed by Zewail and co-workers,^2^ but without the subsequent **S** → **D** step) and **N** → **D** (Takeuchi–Tahara model^4^).

This hypothesis allows to rationalize why there is formation of **S** structures following the Coulomb explosion in the pump–probe measurements by Folmer *et al.*^36^ and—even more puzzling—why the yield of **S** structures increases relative to **D** within the first picosecond after the photoexcitation. The presence of **S** structures has been previously taken as evidence of formation of an intermediate in the stepwise process.^9^,^36^ It has also been attributed to a possibly invasive character of the experimental methodology.^5^ According to our hypothesis, the appearance of **S** structures and even its initial population increase is perfectly compatible with the concerted mechanism, as they should be direct consequence of the **D** → **S** conversion. The picosecond decay of the **S** structures, also reported in ref. 36, may be associated to the internal conversion of **S**, rather than to the **S** → **D** reaction, as formerly proposed.

Unfortunately, there are no gas-phase time-resolved experimental results for 7AI dimer excited in the ballistic (high-energy) region, as in our dynamics simulations. In hexane, 7AI dimer excited in the 270–287 nm range still shows two time-constants, 0.2 and 1 ps.^4^,^35^,^37^ The contribution of the short time-constant relative to that of the long time-constant tends to increase, varying from 1% at 3.96 eV (313 nm) to 35% at 4.43 eV (280 nm).^4^ Due to its apparent invariance upon deuteration, the short time-constant has been assigned to the excited-state relaxation of **N**.^35^ Nevertheless, the systematic rising of the short time-constant contribution with the excitation energy may indicate that the short time-constant indeed signals the ballistic PT process. Moreover, the short time-constant of the deuterated species should increase by only a factor (*M*_D_/*M*_H_)^1/2^ ∼ 1.4 in a ballistic mechanism, which should be below the uncertainty in the transient spectra deconvolution. The formation of **D** structures within ∼0.1 ps, as predicted by our simulations (Table 2), supports the assignment of the 0.2 ps time-constant to the ballistic PT process. This comparison, however, should be taken with reserve due to the differences between the solvated and gas-phase systems.

### 4.2 Critical appraisal of previous simulations

The hypothetical existence of a high-energy **S** intermediate has been a key issue for all previous proposals of a stepwise mechanism. The earlier theoretical models used to rationalize the time-resolved spectroscopy in terms of a stepwise mechanism^2^ (as well as to disproof it^5^), wrongly predicted the existence of a locally-excited (LE) **S** intermediate. Based on the CIS method, they also did not describe the CT state at first. Latter, when the **S** CT structure was finally identified, still using the CIS approach,^38^ it was incorrectly expected to be less stable than **D** (quantitative values for the main topographic features of the S_1_ state computed with diverse methods are given in Table S5 of the ESI†).

This unbalance between **S** CT and **D** in the S_1_ state has been recognized long ago. In particular, the CASPT2 calculations reported in ref. 39 correctly placed **S** CT energetically below **D**. However, those calculations still did not provide a qualitatively correct topography of the excited state. First, they underestimated the vertical excitation at **N** (due to uncorrected zero-order Hamiltonian^40^) and overestimated TS S_1_-N (due to excess of symmetry restrictions), leading to an artificially high barrier for the concerted mechanism. Second, the CASSCF geometry optimizations also predicted an **S** LE intermediate. We discuss in the ESI (Section S10†) that this intermediate, which does not exist on the CC2 surface, is possibly not a minimum but a transition state. This intermediate also does not exist according to other multi-reference perturbation theory (MRMP/CASSCF) simulations.^30^

A topographical analysis of the S_1_ surface with TDDFT based on the LC-BLYP functional favored the concerted mechanism too.^41^ Nevertheless, this method predicted an **S** CT structure slightly above the **D** structure; as a consequence the stepwise mechanism could not be ruled out. The reason for this unbalance was the range-separation parameter employed in the functional, which is not appropriate for describing the 7AI dimer. We show in the ESI (Section S10†) that after a non-empirical re-parametrization of the functional, TDDFT/LC-BLYP renders an **S** CT structure more stable than **D** as well, as predicted by CC2 and CASPT2.^39^

The stability of the **S** CT structure was also recognized in ref. 42 using CIS/TDDFT. Nevertheless, the lack of dispersion corrections led to a dissociative character of the neutral fragments of the CT structure. More recently, Ando *et al.*^30^ provided an essentially correct topography of the excited state using MRMP/CASSCF. That investigation, however, was constrained to a too small area of the Δ*R*_1_ – Δ*R*_2_ plane, not revealing the main features of the CT region: the S_1_ minimum and the S_1_/S_0_ conical intersection.

The present simulations overcome all those previous shortcomings. Our calculations are based on a high-correlated method, able to provide a balanced description of different states; wavefunctions expanded on a large basis set; and geometries and energies computed at the same level with no symmetry restrictions. The main limitations of the present approach, single reference ground states and approximated double excitations, have been evaluated using different diagnostic tools and do not pose any problems for the structures investigated here (ESI, Section S11†).

## 5. Conclusions

Although there are compelling experimental evidences favoring the concerted mechanism in the photoinduced double PT in 7AI dimer in the gas phase,^11^ the stepwise mechanism could never been really ruled out. The main reason for this ambiguity is that all computational simulations of 7AI dimer so far failed, in a way or other, to describe some key features of the excited-state potential energy surface, thus leading to biased discussions. In this work, we have provided a description of the excited-state potential energy surface computed with a high-level *ab initio* theory, adequate to treat different adiabatic characters of the excited-state surface in a balanced way.

Based on these calculations, we show that the stepwise mechanism is not consistent with the topography of the excited state. This topography clearly reveals that if a single-PT structure is formed (either *via* tunneling or a ballistic process), it will be more stable than a double-PT structure and will quickly undergo internal conversion to the ground state. Therefore, the excited-state stepwise mechanism is kinetically and thermodynamically unfavorable in the gas phase.

The topographical analysis also points out to a split of the population between: (a) dimers undergoing tunneling into single-PT and double-PT structures (slow mechanism); and (b) dimers undergoing asynchronous concerted (ballistic or near-edge tunneling) double PT (fast mechanism). This population split, which should be deeply dependent on the excitation energy and solvation conditions, is likely the origin of the two time constants observed in time-resolved experiments. Independently of their formation mechanism, when double-PT structures arise, they either convert into CT structures or decay *via* fluorescence; when CT structures arise, they decay to the ground state *via* internal conversion. This working hypothesis still needs to be corroborated by simulations incorporating tunneling and isotopic effects based on potential energy surfaces owing the correct topography.

Time-resolved spectroscopic measurements often result in highly convoluted data, which require a number of theoretical hypotheses to treat and interpret them.^43^ For this reason, the synergy between these experimental techniques and computational-chemistry simulations has been extremely positive. We should be aware, however, that, due to computational costs and conceptual difficulties,^44^ computational simulations of excited states are usually based on strong approximations. The case of 7AI dimer presented here raises a warning flag of how such approximations may render qualitatively incorrect pictures, leading to unphysical interpretation of experimental data.

## Supplementary Material

Supplementary information
