# Supplementary material for: Stepwise double excited-state proton transfer is not possible in 7-azaindole dimer
Source: Chem Sci. 2015 Jul 8;6(10):5762–7. doi: 10.1039/c5sc01902h (PMC5947511; doi:10.1039/c5sc01902h)
Supplement: Supplementary file 1 [file SC-006-C5SC01902H-s001.pdf]

Electronic Supplementary Material (ESI) for Chemical Science.
This journal is © The Royal Society of Chemistry 2015

ELECTRONIC SUPPLEMENTARY INFORMATION - ESI

Stepwise Double Excited-State Proton Transfer Is Not Possible in 7-Azaindole

Rachel Crespo-Otero, Nawee Kungwan, Mario Barbatti

LIST OF CONTENTS

SECTION S1. SURVEY OF EXPERIMENTAL DATA 2
SECTION S2. COMPUTATIONAL METHODS 4
SECTION S3. GEOMETRIES 6
SECTION S4. VERTICAL EXCITATIONS AND ABSORPTION SPECTRUM 8
SECTION S5. ENERGIES AND OSCILLATOR STRENGTHS 9
SECTION S6. THE $\Delta R_1 - \Delta R_2$ PLANE 12
SECTION S7. EXAMPLES OF TRAJECTORIES 14
SECTION S8. TIME EVOLUTION OF TRAJECTORIES 16
SECTION S9. ANALYSIS OF SPT FORMATION AND INTERNAL CONVERSION 17
SECTION S10. ANALYSES OF PREVIOUS RESULTS 18
SECTION S11. MULTIREFERENCE AND DOUBLE EXCITATIONS 20
SECTION S12. ADDITIONAL REFERENCES 22
SECTION S13. CARTESIAN COORDINATES 24

SECTION S1. SURVEY OF EXPERIMENTAL DATA

Table S1. Survey of experimental results for the excited-state time-constants of 7AI dimer obtained using different experimental techniques and conditions. Long time constants (>10 ps) are not reported here, neither are results for deuterated species.

τ_1 (fs)	τ_2 (ps)	Pump (nm)	Probe/Observ. (nm)	Technique	Medium	Year[Ref.]
650<br>360<br>200	3.3<br>1.7<br>1.6	310<br>307<br>305	620<br>620<br>620	Femtosecond Resolved Mass Spectroscopy (236 amu); Pulse: 60 fs, 0.5 mJ	Gas Phase	1995[1]
-	<< 5	310	310	Time Resolved Photoelectron Spectroscopy; Pulses: 0.8 ps, 0.5 ps	Gas Phase	1997[2]
660	5.0	312	624	Time Resolved Coulomb Explosion; Detection: Ratio 119 amu /118 amu; Pulse: 120 fs, 2 mJ	Gas Phase	1998[3, 4]
-	~2.0	0-0	-	Disperse Fluorescence (Frequency-domain)	Gas Phase	2005[5]
-<br>-	1.9<br>0.86	0-0<br>1σ	620<br>620	Picosecond Resolved Resonance Enhanced Multiphoton Ionization; Pulse: 2.7 ps	Gas Phase	2005[6]
-	<< 5	263.5	450-560	Time Resolved Fluorescence Spectroscopy; Pulse: 3-6 ps, 0.1 mJ	3-Methylpentane	1979[7]
-	1.4	608	480	Femtosecond Fluorescence Up-Conversion; Pulse: 250 fs, 400 μJ	Hexadecane	1991[8]
-	1.4	312 [9]	375, 500	Transient Absorption		
200	1.1	270	320-620	Femtosecond Fluorescence Up-Conversion; Pulse: 280 fs	Hexane	1997[10, 11]
200<br>-	1.0<br>1.0	306<br>306	360<br>480	Femtosecond Fluorescence Up-Conversion; Pulse: 90 fs, 0.6 mJ	Hexadecane<br>3-Methylpentane	1998[12]
130	1.2	320	394-460	Femtosecond Time Resolved Absorption; Pulse: 90 fs, 0.6 mJ	3-Methylpentane	1999[13]
-	~1.0	266	360-480	Femtosecond Fluorescence Up-Conversion; Pulse: 90 fs, 0.6 mJ		
550	2.0-2.8	310	620	Time Resolved Coulomb Explosion; Detection: Ratio 119 amu /118 amu; Pulse: 200 fs, 1.5 mJ	Water clusters	1999[14]

200	1.1	280,287	380	Femtosecond	Hexane	2007[15]
-	1.1	313	380	Fluorescence Up-Conversion; Pulse: 290 fs		
-	1.1	313	380	Femtosecond Time Resolved Fluorescence	n-Heptane, n-Dodecane, n-Hexadecane	2007[16]
-	2.2	313	380		Acetonitrile	

SECTION S2. COMPUTATIONAL METHODS

Excited states were computed with the coupled cluster to approximated second order (CC2)[17-19] and with the algebraic diagrammatic construction to the second order [ADC(2)],[20, 21] both using the resolution-of-the-identity (RI) approximation.[19] In the case of the ADC(2), the corresponding ground state was computed at the second-order Møller-Plesset Perturbation Theory (MP2).[18] CC2 calculations were done with the TZVP basis set.[22] ADC(2) calculations were done with the SV(P) and TZVP basis sets. Frozen 1s cores were used in both, CC2 and ADC(2), during geometry optimizations and single point calculations. Additional calculations were done with the time-dependent functional theory (TDDFT), using the long-range corrected functional LC-BLYP.[23-25]

Transition states were computed in the excited states based on numerical Hessians. Due to the high computational costs of these calculations, the Hessians were always computed with ADC(2)/SV(P). The Hessians were also always recomputed after optimization was reached. Conical intersections were optimized with the penalty Lagrange multiplier technique (α = 0.02 Hartree) implemented in the CIOPT program,[26] which we have adapted to work with CC2 and ADC(2).

Exploratory dynamics simulations in the excited states were also computed. First, the absorption spectrum was simulated at the ADC(2)/SV(P) level with the nuclear ensemble method.[27] Initial conditions were sampled from two energy windows in the spectrum: 4.1±0.1 eV (A) and 4.7±0.1 eV (B). The initial states were determined according to the distribution of oscillator strengths within each window. In window A, 20 trajectories were initiated in S_1. In window B, 7 trajectories were initiated in S_2, 12 in S_3 and 5 in S_4, in a total of 24 trajectories. Due to the reduced number of trajectories, all dynamics results have low statistical significance and they should be understood as a qualitative exploration of the potential surfaces. For 90% confidence interval, all reported yields have maximum statistical uncertainties of ±18%.

On-the-fly dynamic simulations were carried out on the excited states computed with the RI-ADC(2)/SV(P) level of theory. All electrons, including the 1s cores, were taken into account. Starting in window A, only the S_1 state was considered. Starting in window B, all excited states up to S_4 were included. Non-adiabatic effects were taken into account by the surface hooping approach.[28] Classical equations were integrated with 0.5 fs time step, while quantum equations were integrated with 0.025 fs using interpolated quantities between classical steps. The maximum simulation time was 1000 fs. Hopping probabilities between excited states were computed with the fewest switches approach[29] including decoherence corrections (α = 0.1 Hartree).[30]

Nonadiabatic couplings with ADC(2) were computed by finite differences with the method discussed in Ref.[31] based on the Hammes-Schiffer-Tully approach.[32] As a single reference method, ADC(2) cannot provide reliable nonadiabatic couplings for crossings with the ground state.[31] For this reason, when a trajectory reached an S_1-S_0 energy gap smaller than 0.1 eV before the maximum simulation time (1 ps), it was stopped. This crossing time was taken as an estimate of the internal conversion time to the ground state.

As usual in surface hopping,[33] no tunneling processes were computed. For the above-the-barrier excitations considered here, this is a minor approximation as ballistic processes should dominate the excited state relaxation.

CC2 and ADC(2) calculations were carried out with the TURBOMOLE program.[34] The spectrum and dynamics simulations were performed with NEWTON-X[35, 36] interfaced with TURBOMOLE. TDDFT calculations were done with Gaussian 09.[37]

SECTION S3. GEOMETRIES

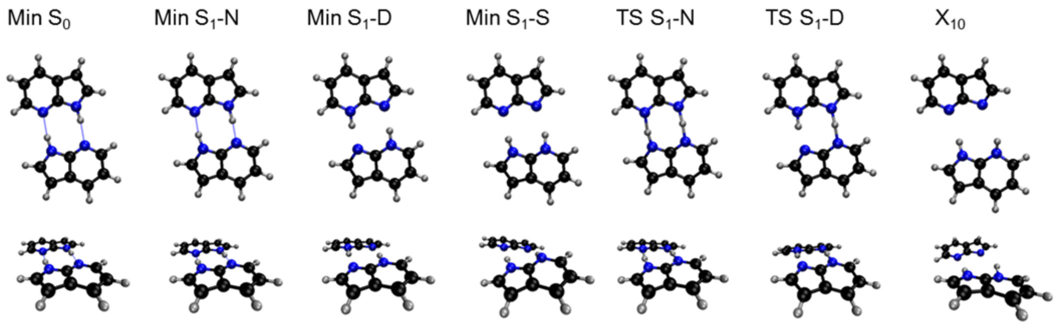

Fig. S1. Geometry of stationary points and conical intersection. Ground and excited state minima (Min), transition states (TS) on the S_1 state and conical intersections (X) between S_0 and S_1 were optimized with CC2/TZVP, ADC(2)/TZVP and ADC(2)/SV(P) levels. Their Cartesian coordinates are given at the end of this document.

Table S2. Proton transfer coordinates for stationary structures and conical intersections computed at diverse levels.

ADC(2)/SV(P)						
Geometry	R^{α}_{N1H} (Å)	R^{β}_{N6H} (Å)	R^{β}_{N1H} (Å)	R^{α}_{N6H} (Å)	ΔR_1 (Å)	ΔR_2 (Å)
Min S_0	1.043	1.884	1.043	1.885	-0.84	-0.84
Min S_1-N	1.085	1.681	1.072	1.698	-0.60	-0.63
Min S_1-S	2.161	1.024	1.024	2.119	1.14	-1.10
Min S_1-D	1.830	1.054	1.829	1.050	0.78	0.78
X_{10}	2.834	1.018	1.014	2.442	1.82	-1.43
TS S_1-N	1.261	1.335	1.157	1.472	-0.07	-0.32
TS S_1-D	1.198	1.402	1.797	1.055	-0.20	0.74
Monomer S_1	1.020	∞	1.020	∞	-∞	-∞
ADC(2)/TZVP						
Geometry	R^{α}_{N1H} (Å)	R^{β}_{N6H} (Å)	R^{β}_{N1H} (Å)	R^{α}_{N6H} (Å)	ΔR_1 (Å)	ΔR_2 (Å)
Min S_0	1.033	1.879	1.033	1.879	-0.85	-0.85
Min S_1-N	1.074	1.671	1.059	1.701	-0.60	-0.64
Min S_1-S	2.169	1.018	1.015	2.104	1.15	-1.09
Min S_1-D	1.825	1.043	1.818	1.041	0.78	0.78
X_{10}	2.857	1.012	1.007	2.446	1.85	-1.44
TS S_1-N	1.260	1.312	1.126	1.498	-0.05	-0.37
TS S_1-D	1.198	1.371	1.785	1.046	-0.17	0.74
Monomer S_1	1.012	∞	1.012	∞	-∞	-∞

CC2/TZVP						
Geometry	R^{α}_{N1H} (Å)	R^{β}_{N6H} (Å)	R^{β}_{N1H} (Å)	R^{α}_{N6H} (Å)	ΔR_1 (Å)	ΔR_2 (Å)
Min S_0	1.037	1.867	1.037	1.867	-0.83	-0.83
Min S_1-N	1.082	1.653	1.065	1.689	-0.57	-0.62
Min S_1-S	2.157	1.020	1.017	2.093	1.14	-1.08
Min S_1-D	1.814	1.046	1.809	1.044	0.77	0.77
X_{10}	2.883	1.019	1.009	2.459	1.86	-1.45
TS S_1-N	1.249	1.328	1.132	1.491	-0.08	-0.36
TS S_1-D	1.197	1.376	1.778	1.049	-0.18	0.73
Monomer S_1	1.013	∞	1.013	∞	-∞	-∞
CASSCF(12,12)/6-31G(d,p)[38]						
Geometry	R^{α}_{N1H} (Å)	R^{β}_{N6H} (Å)	R^{β}_{N1H} (Å)	R^{α}_{N6H} (Å)	ΔR_1 (Å)	ΔR_2 (Å)
Min S_0 (AD_{S0})	1.002	2.142	1.002	2.142	-1.14	-1.14
Min S_1-N (AD_{S1})	1.004	2.108	1.005	2.072	-1.10	-1.07
Min S_1-S (DI_N)	2.307	0.995	0.996	2.331	1.31	-1.34
Min S_1-D (TD_{S1})	2.098	1.006	2.119	1.000	1.09	1.12
TS S_1-N (DI_{TS})	1.232	1.325	1.232	1.325	-0.09	-0.09
DI_I	1.018	1.889	1.103	1.558	-0.87	-0.46

SECTION S4. VERTICAL EXCITATIONS AND ABSORPTION SPECTRUM

Table S3. Vertical excitation energies and oscillator strengths of the C_{2h} 7AI dimer at the S_0 minimum computed with different methods.

	ADC(2)/SV(P)		ADC(2)/TZVP		CC2/TZVP		
	ΔE (eV)	*f*	ΔE (eV)	*f*	ΔE (eV)	*f*	Assignment
S_1	4.667	0.112	4.568	0.112	4.577	0.100	ππ* (B_u)
S_2	4.688	0.000	4.586	0.000	4.594	0.000	ππ* (A_g)
S_3	4.880	0.000	4.778	0.000	4.777	0.000	ππ* (A_g)
S_4	4.907	0.228	4.808	0.241	4.807	0.208	ππ* (B_u)
S_5			5.515	0.000	5.544	0.000	nπ*+ππ*
S_6			5.519	0.005	5.547	0.005	nπ*+ππ*
S_5	6.266	0.000					ππ* (A_g)
S_6	6.395	0.142					ππ* (B_u)

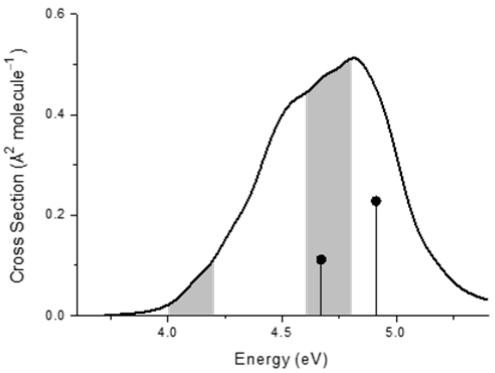

Fig. S2. 7AI dimer absorption spectrum simulated with the nuclear ensemble method.[27] A Wigner distribution with N_p = 500 points was generated around the C_{2h} S_0 minimum structure. N_{fs} = 6 excited states were included. The convolution was done with Gaussian functions with δ = 0.1 eV width. Excited states were computed with ADC(2)/SV(P). The shaded areas indicate the two windows from where initial conditions were selected, 4.1±0.1 eV (A) and 4.7±0.1 eV (B). Dots indicate the vertical excitation energies and oscillator strengths of the first two bright excited states (S_1 and S_4).

SECTION S5. ENERGIES AND OSCILLATOR STRENGTHS

Table S4. Energies and oscillator strengths for stationary structures and conical intersections computed at diverse levels.

		ADC(2)/SV(P)					
Geometry	S_0 (au)	S_1 (au)	*f*	S_0 (eV)	S_1 (eV)	ΔE (eV)	λ (nm)
Min S_0	-756.742421	0.171521	0.112	0.000	4.667	4.67	266
Min S_1-N	-756.724052	0.138362	0.078	0.500	4.265	3.77	329
Min S_1-S	-756.671753	0.050461	0.001	1.923	3.296	1.37	903
Min S_1-D	-756.697126	0.091062	0.019	1.233	3.710	2.48	500
X_{10}	-756.614386	0.000724	-	3.484	3.504	0.02	-
TS S_1-N	-756.707481	0.124556	-	0.951	4.340	3.39	-
TS S_1-D	-756.696712	0.111762	-	1.244	4.285	3.04	-
Monomer S_1	-756.694002	0.165250	0.085	1.318	5.814	4.50	276
		ADC(2)/TZVP					
Geometry	S_0 (au)	S_1 (au)	*f*	S_0 (eV)	S_1 (eV)	ΔE (eV)	λ (nm)
Min S_0	-757.770212	0.167877	0.112	0.000	4.568	4.57	271
Min S_1-N	-757.750926	0.132584	0.074	0.525	4.133	3.61	344
Min S_1-S	-757.696095	0.042071	0.000	2.017	3.162	1.14	1083
Min S_1-D	-757.722914	0.084709	0.016	1.287	3.592	2.31	538
X_{10}	-757.648602	0.000675	-	3.309	3.328	0.02	-
TS S_1-N	-757.735055	0.119243	-	0.957	4.201	3.24	-
TS S_1-D	-757.721539	0.103277	-	1.324	4.135	2.81	-
Monomer S_1	-757.720380	0.158643	0.102	1.356	5.673	4.32	287
		CC2/TZVP					
Geometry	S_0 (au)	S_1 (au)	*f*	S_0 (eV)	S_1 (eV)	ΔE (eV)	λ (nm)
Min S_0	-757.810311	0.168186	0.100	0.000	4.577	4.58	271
Min S_1-N	-757.791186	0.133108	0.062	0.520	4.142	3.62	342
Min S_1-S	-757.739103	0.046988	0.002	1.938	3.216	1.28	970
Min S_1-D	-757.767061	0.089123	0.015	1.177	3.602	2.43	511
X_{10}	-757.686573	0.001358	-	3.367	3.404	0.04	-
TS S_1-N	-757.777423	0.121139	-	0.895	4.191	3.30	-
TS S_1-D	-757.764932	0.106737	-	1.235	4.139	2.90	-
Monomer S_1	-757.760249	0.159562	0.102	1.362	5.704	4.34	286
		CASPT2(16,14)/ANO[38]					
Geometry	S_0 (au)	S_1 (au)	*f*	S_0 (eV)	S_1 (eV)	ΔE (eV)	λ (nm)
Min S_0 (AD_{S0})				0.000	4.020	4.02	308
Min S_1-N (AD_{S1})				0.180	3.870	3.69	336
Min S_1-S (DI_N)				2.080	3.270	1.19	1042
Min S_1-D (TD_{S1})				1.444	3.594	2.15	577

Geometry	S_0 (au)	S_1 (au)	*f*	S_0 (eV)	S_1 (eV)	ΔE (eV)	λ (nm)
TS S_1-N (DI_{TS})					4.520		
DI_I					4.27		
			Expt.[5, 15]				
Geometry	S_0 (au)	S_1 (au)	*f*	S_0 (eV)	S_1 (eV)	ΔE (eV)	λ (nm)
Min S_0						4.23	293
Min S_1-N						3.47	357
Min S_1-S						-	-
Min S_1-D						2.48	500

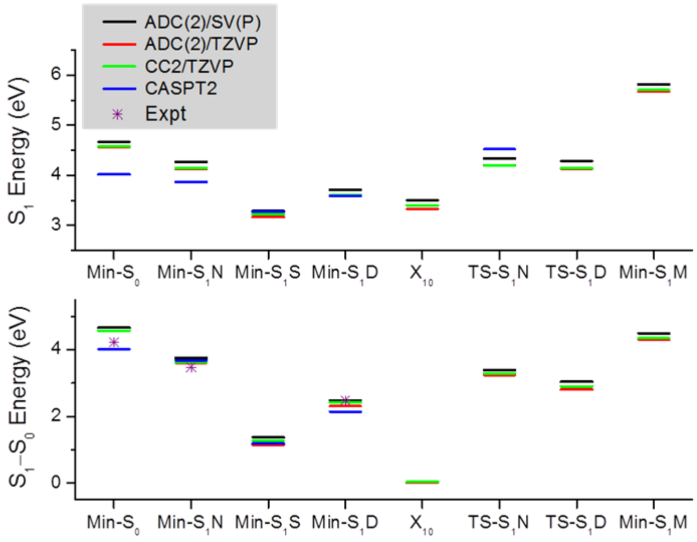

Fig. S3. Comparison of energies computed with different methods. CASPT2 results from Ref.[38].

Table S5. Potential energy (eV) of the main features of the S_1 state computed with diverse methods, taking the S_1 energy of the Min S_1-N as the reference.

Method	Min S_1-N	Min S_1-S (CT)	Min S_1-S (LE)	Min S_1-D	X_{10}	TS S_1-N	TS S_1-D
CC2 (This work)	0.00	-0.93	-	-0.54	-0.74	0.05	0.00
CASPT2(16,14)[38]	0.00	-0.60	0.40	-0.28	-	0.65	-
MRMP(8,8)[39]	0.00	-	-	-0.23	-	0.19	-
TD-LCBYLP[40]	0.00	-0.21	-	-0.30	-	0.07	0.26
CIS[41]	0.00	-0.06	0.68	-0.18	-	0.75	0.69

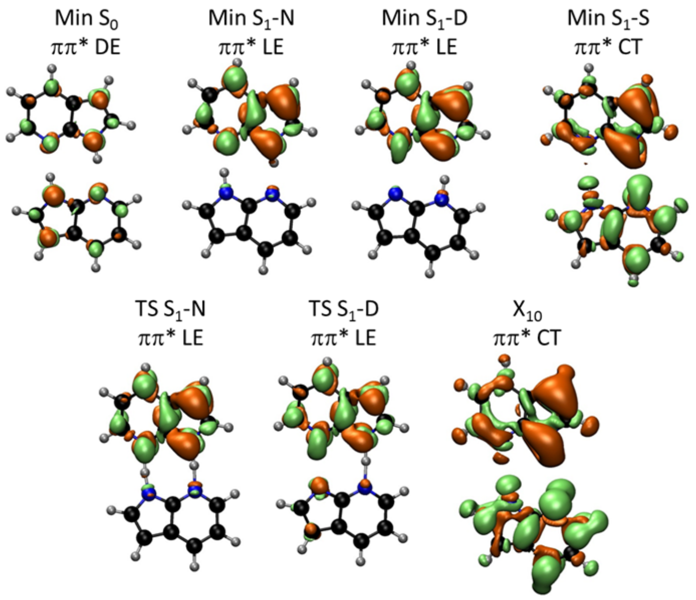

Fig. S4. S_1-S_0 Difference densities for all stationary points and conical intersection computed at CC2/TZVP. Orange – electron donor; green – electron acceptor. Based on these densities, states can classified as delocalized (Deloc), localized (Loc), or charge transfer (CT).

SECTION S6. THE ΔR_1 – ΔR_2 PLANE

The proton transfers in 7AI dimer can be conveniently discussed in terms of the ΔR_1–ΔR_2 plane defined by the internal coordinates

$$\Delta R_1 = R^{\alpha}_{N1H} - R^{\beta}_{N6H},$$

$$\Delta R_2 = R^{\beta}_{N1H} - R^{\alpha}_{N6H},$$

where R^{m}_{N1H} is the NH distance in the pyrrole group of monomer *m* and R^{n}_{N6H} is the NH distance in the pyridine group of monomer *n*. (Fig. S5-left). All stationary structures and conical intersections discussed in this work can be directly visualized in this plane (Fig. S5-center; see also Table S2).

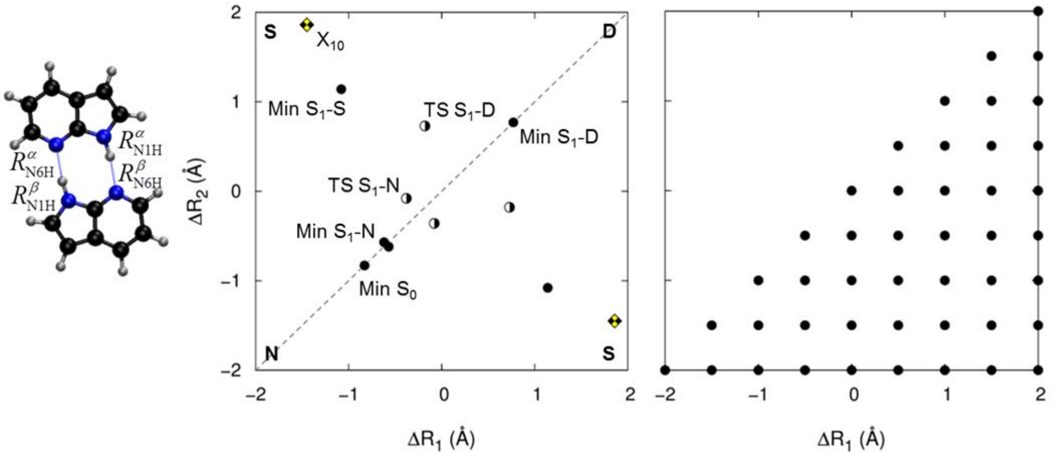

Fig. S5. (Left) Definition of internal coordinates. (Center) Stationary points and conical intersections are indicated by points on the ΔR_1–ΔR_2 plane. Structures are symmetric by reflection at the dashed line. The four quadrants are named **N** (normal), **S** (single PT), and **D** (double PT) according to the geometry of the tautomers in that region. (Right) The points indicate the 45 structures optimized in the S_1 state. The upper half of the plane was obtained by symmetry, providing a total of 81 points.

The S_1 geometries of 45 points on the ΔR_1–ΔR_2 plane were optimized by freezing ΔR_1 and ΔR_2 and relaxing all other coordinates. These optimizations were carried out first at ADC(2)/SV(P) level, and later refined at CC2/TZVP level. Excitation energies into the S_1 state at both levels are shown in Fig. S6. There is a strong quantitative agreement between CC2 and ADC(2) energies. For further comparison between the two levels, see Section S5.

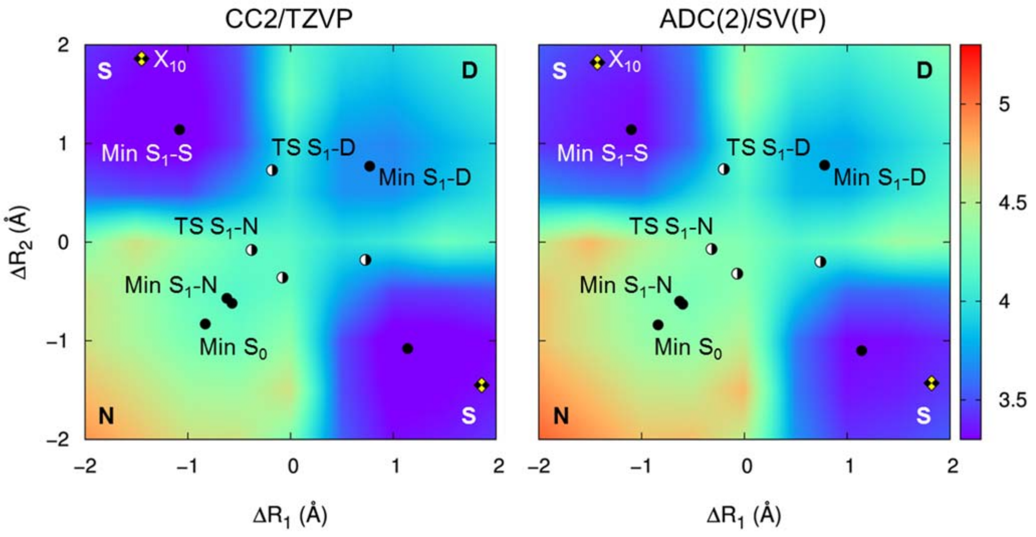

Fig. S6. Excitations energies (eV) into the S_1 state. 81 optimized structures were included in each graph.

SECTION S7. EXAMPLES OF TRAJECTORIES

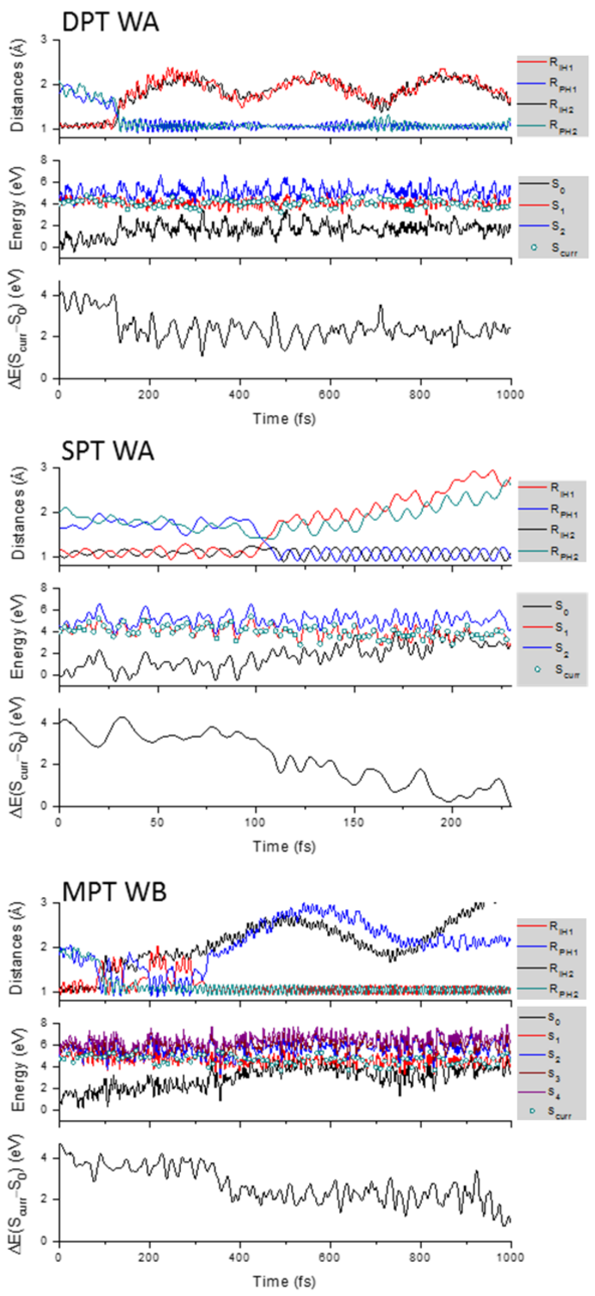

Fig. S7. Evolution of geometry and energies along three selected trajectories. Top: DPT (double proton transfer) is the most common case in both excitation windows. After the proton transfers at ~120 fs, an oscillation with 500 fs period appears in the R_{IH} coordinates, reflecting the inter-monomer vibrations. Middle: SPT (single proton transfer) is quantitatively less important. After its occurrence, the trajectory tends to quickly find an intersection with S_0. Bottom: MPT (multiple proton transfer) trajectories always

start as a DPT, but an additional transfer occurs forming an S structure. As the SPT trajectories, after formation of S, the energy gap with S_0 tends to decrease towards the intersection.

SECTION S8. TIME EVOLUTION OF TRAJECTORIES

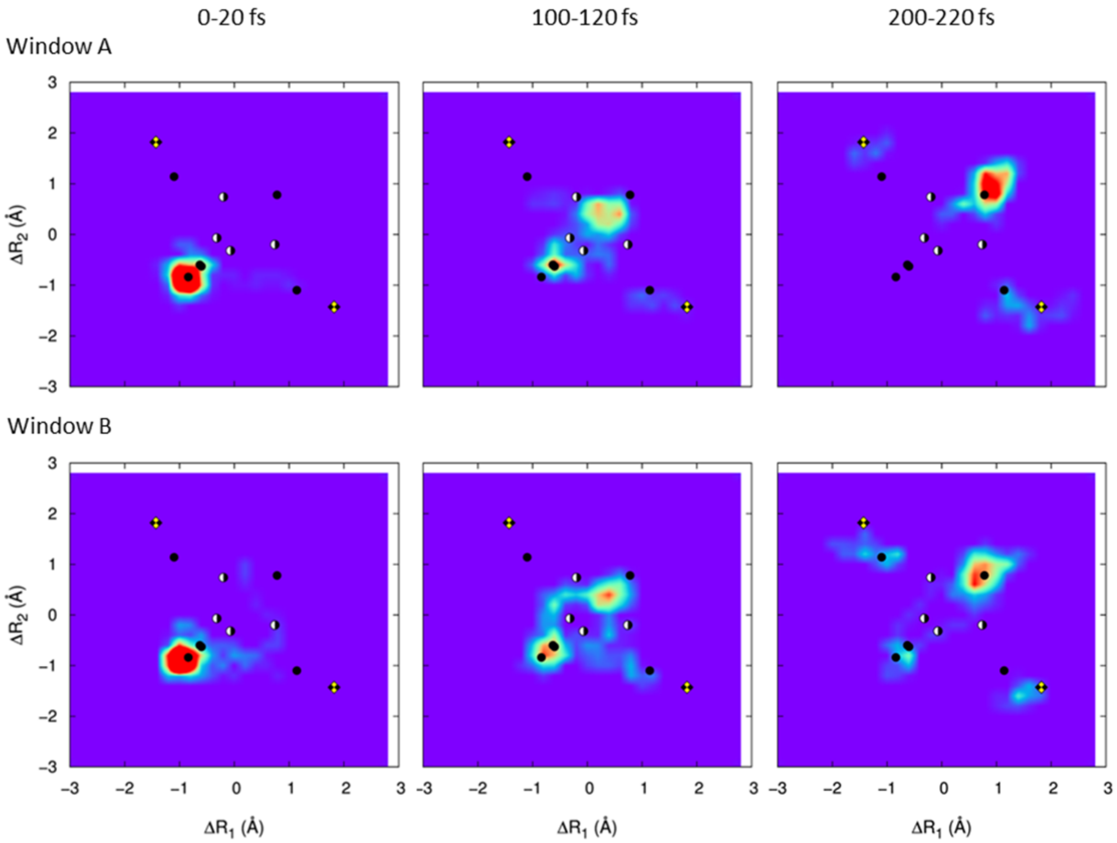

Fig. S8. Trajectory distribution in three different time intervals, 0-20 fs, 100-120 fs, and 200-220 fs, in window A (top) and B (bottom). Density grows from violet to red. Initially (left), the dimer is located at the **N** region. After 100 fs (center), trajectories are distributed around the diagonal connection **N** and **D** indicating the occurrence of asynchronous DPT. At 200 fs (right) most of population is at the **D** region, with a minor fraction at the **S** region.

SECTION S9. ANALYSIS OF SPT FORMATION AND INTERNAL CONVERSION

Table S6. Characterization of the of single proton transfer structures formed in SPT and MPT trajectories. τ_{SPT} is the time when the trajectory crosses to the single PT structure. τ_{X10} is the time when the trajectory reaches the intersection with the ground state. If the intersection is not reached within 1 ps, the energy gap at this time is shown. In average, the crossing is reached 140±68 fs after the formation of the SPT structure. Three trajectories did not find the crossing with the ground state within 1 ps. Two of them ended with energy gaps below 1 eV, indicating that the crossing should be near. The third one ended with a large energy gap (2.3 eV). In this case, however, the SPT structure was formed at 991 fs, and the trajectory did not have enough time to relax.

Window	Type	Traj	τ_{SPT} (fs)	τ_{X10} (fs)
WA	SPT	1	118	238
		2	11	237
		3	105	230
	MPT	1	130	259
WB	SPT	1	89	240
		2	12	187
	MPT	1	991	[2.3 eV]
		2	123	301
		3	317	563
		4	119	310
		5	684	723
		6	345	[0.97 eV]
		7	153	257
		8	408	424
		9	926	[0.82 eV]

SECTION S10. ANALYSES OF PREVIOUS RESULTS

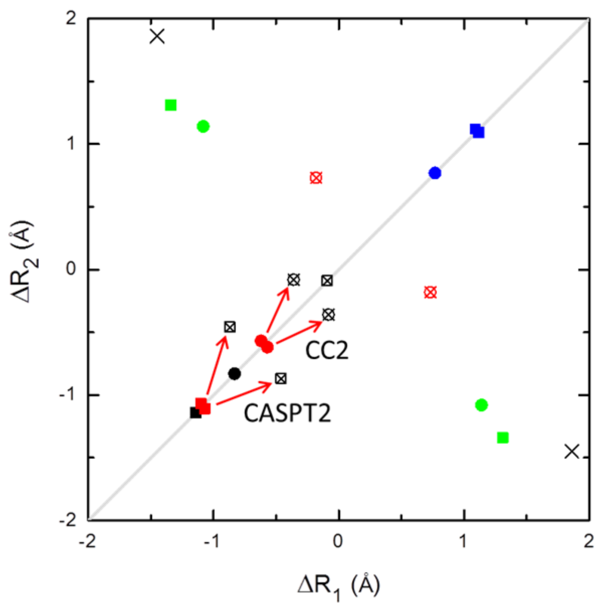

Fig. S9. Stationary points and conical intersections on the S_1 state. Circles indicate CC2 results (present work) and squares indicate CASSCF geometries used for CASPT2 calculations in Ref.[38]. The crosses are the conical intersections with S_0. Inclusion of dynamic correlation in CC2 tends to displace all stationary points to central regions of the plane, as compared to CASSCF. The arrows in the CC2 results indicate the relation between the S_1 minimum N (red circles) and the transition state I (crossed black circle). A similar set of arrows for CASSCF connects the S_1 minimum N (red squares) to another S_1 minimum (black crossed squares), which is claimed to be the intermediate to stepwise reactions.[38] Such intermediate, however, does not exist on the CC2 and ADC(2) surfaces. From the topographic relations in this figure, there is the possibility that the claimed intermediate is in fact a transition state, as they cannot be distinguished from minima during simple optimization procedures. Moreover, even if this intermediate minimum existed, its proximity to the main diagonal in the plane would make it more an intermediate for concerted reactions than for stepwise reactions.

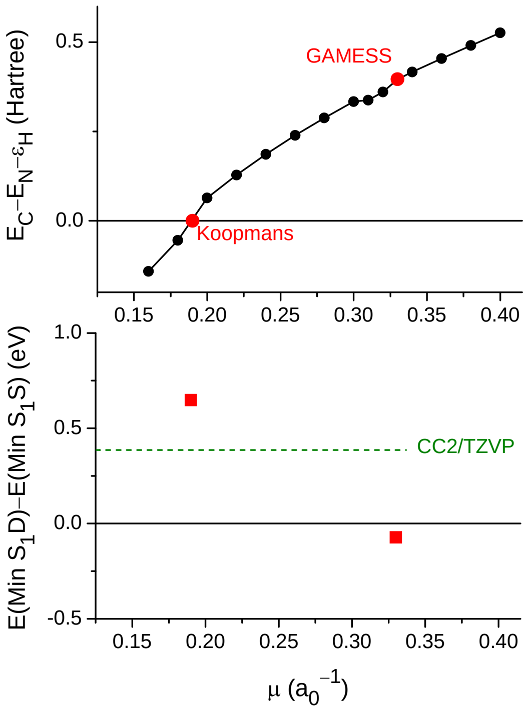

Fig. S10. Performance of TDDFT with the LC-BLYP functional. When LC-BLYP is used with a range-separation parameter of 0.33 a_0^{-1} as recommended in Ref.[42] and implemented in GAMESS,[43] the S_1-excited S structure is slightly less stable than the D structure by 0.07 eV (Bottom graph). Accordingly, in Ref.[40], where GAMESS was used, S was less stable than D by 0.09 eV. A non-empirical parameterization of the range-separation parameter following the method discussed in Ref.[44] (Top graph) indicates that a more adequate value for computing 7AI dimer is instead μ = 0.19 a_0^{-1}. With this value, the S structure is more stable than the D structure by 0.65 eV, in qualitative agreement with the CC2 result (0.39 eV).

SECTION S11. MULTIREFERENCE AND DOUBLE EXCITATIONS

As single-reference methods with approximated doubles, CC2 and ADC(2) may not perform well when either the ground state has multireference character or the excited state has large contributions from double excitations. To evaluate the present results for these potential shortcomings, we have computed the D_1, D_2, and $\%\tau_2$ diagnostics. The D_1 diagnostic[45] measures the interaction between the Hartree-Fock (HF) reference state and singly-excited determinants. Large values of D_1 indicate multireference character of the ground state. Analogously, the D_2 diagnostic[46] measures the interaction between the HF reference and doubly-excited determinants, thus, high values indicate large contributions of double excitations to the ground state. $\%\tau_2$ measures the contribution of double excitations to a particular excited state.[47] (For $\%\tau_2$, we have used the new definition implemented in Turbomole starting from version 6.5.)

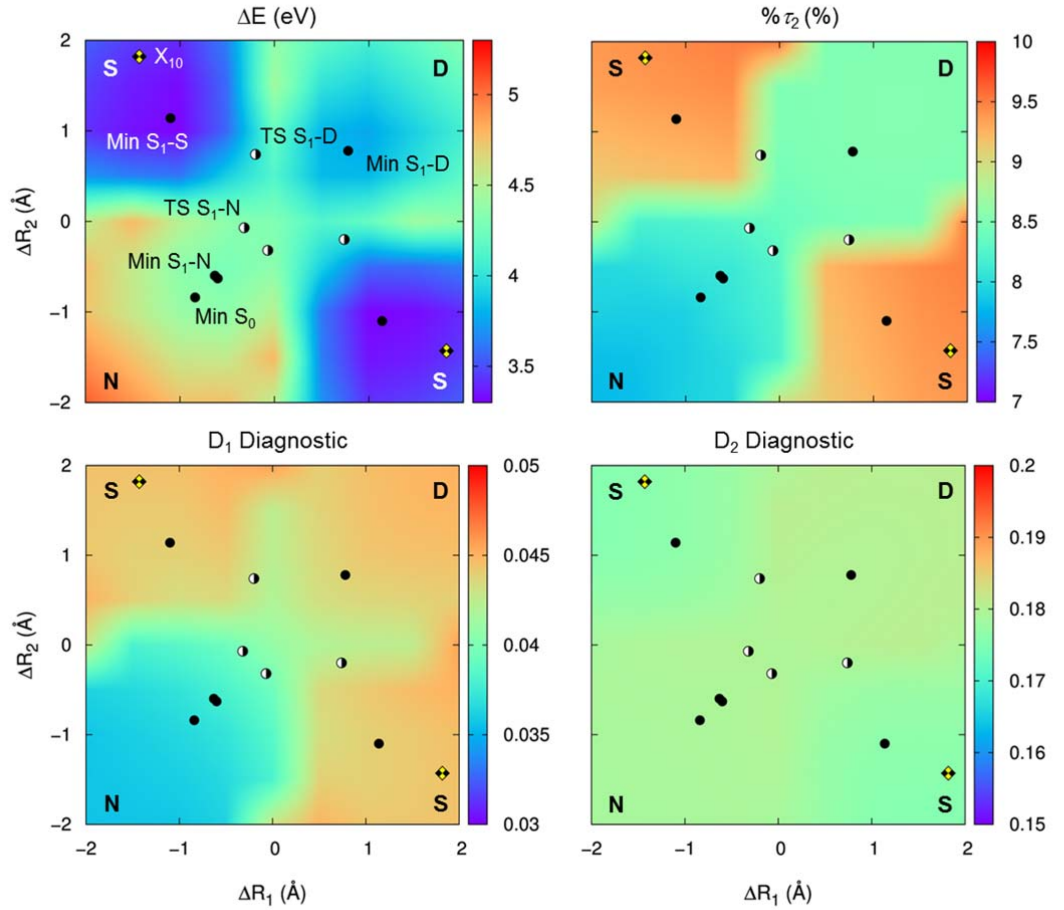

Fig. S11. ΔE, $\%\tau_2$, D_1 and D_2 for ADC(2)/SV(P) on the ΔR_1-ΔR_2 plane. Stationary points and conical intersections are indicated as well.

D_1, D_2 and $\%\tau_2$ diagnostics were computed for all points on the (relaxed) ΔR_1-ΔR_2 plane. D_1 and D_2 were obtained at the MP2/SV(P) level (MP2 is the reference ground state for ADC(2)), while $\%\tau_2$ was

computed at ADC(2)/SV(P) level for the S_1 state. Because the three diagnostics were computed with the lowest computational level used in this work, the values discussed here should provide the upper limit for these quantities.

The D_1 and D_2 values are shown in Fig. S11. D_1 is about 0.035 in the **N** region and increases to 0.045 in the **D** and **S** regions. D_2 is about 0.18 in the whole plane. These values are within normal thresholds, indicating that multireference character does not pose a problem.

$\%\tau_2$ is 0.08 in the N and D regions and 0.095 in the S region. Such values are much smaller than the maximum recommended threshold, 0.15. Therefore, double excitations are also not a problem for the description of the S_1 state of the current structures.

SECTION S12. ADDITIONAL REFERENCES

1. A. Douhal, S. K. Kim and A. H. Zewail, *Nature*, 1995, **378**, 260-263.
2. R. Lopez-Martens, P. Long, D. Solgadi, B. Soep, J. Syage and P. Millie, *Chem. Phys. Lett.*, 1997, **273**, 219-226.
3. D. E. Folmer, L. Poth, E. S. Wisniewski and A. W. Castleman Jr, *Chem. Phys. Lett.*, 1998, **287**, 1-7.
4. D. E. Folmer, E. S. Wisniewski and A. W. Castleman Jr, *Chem. Phys. Lett.*, 2000, **318**, 637-643.
5. K. Sakota and H. Sekiya, *J. Phys. Chem. A*, 2005, **109**, 2722-2727.
6. K. Sakota, C. Okabe, N. Nishi and H. Sekiya, *J. Phys. Chem. A*, 2005, **109**, 5245-5247.
7. W. M. Hetherington III, R. H. Micheels and K. E. Eisenthal, *Chem. Phys. Lett.*, 1979, **66**, 230-233.
8. P. Share, M. Pereira, M. Sarisky, S. Repinec and R. M. Hochstrasser, *J. Lumin.*, 1991, **48–49**, 204-208.
9. S. Abrash, S. Repinec and R. M. Hochstrasser, *J. Chem. Phys.*, 1990, **93**, 1041-1053.
10. S. Takeuchi and T. Tahara, *Chem. Phys. Lett.*, 1997, **277**, 340-346.
11. S. Takeuchi and T. Tahara, *J. Phys. Chem. A*, 1998, **102**, 7740-7753.
12. M. Chachisvilis, T. Fiebig, A. Douhal and A. H. Zewail, *J. Phys. Chem. A*, 1998, **102**, 669-673.
13. T. Fiebig, M. Chachisvilis, M. Manger, A. H. Zewail, A. Douhal, I. Garcia-Ochoa and A. de La Hoz Ayuso, *J. Phys. Chem. A*, 1999, **103**, 7419-7431.
14. D. E. Folmer, E. S. Wisniewski, S. M. Hurley and A. W. Castleman, Jr., *Proc. Natl. Acad. Sci. USA*, 1999, **96**, 12980-12986.
15. S. Takeuchi and T. Tahara, *Proc. Natl. Acad. Sci. USA*, 2007, **104**, 5285-5290.
16. O.-H. Kwon and A. H. Zewail, *Proc. Natl. Acad. Sci. USA*, 2007, **104**, 8703-8708.
17. O. Christiansen, H. Koch and P. Jorgensen, *Chem. Phys. Lett.*, 1995, **243**, 409-418.
18. C. Hättig and F. Weigend, *J. Chem. Phys.*, 2000, **113**, 5154-5161.
19. C. Hättig and A. Köhn, *J. Chem. Phys.*, 2002, **117**, 6939-6951.
20. J. Schirmer, *Phys. Rev. A*, 1982, **26**, 2395-2416.
21. A. B. Trofimov and J. Schirmer, *J. Phys. B: At., Mol. Opt. Phys.*, 1995, **28**, 2299-2324.
22. A. Schäfer, C. Huber and R. Ahlrichs, *J. Chem. Phys.*, 1994, **100**, 5829-5835.
23. H. Iikura, T. Tsuneda, T. Yanai and K. Hirao, *J. Chem. Phys.*, 2001, **115**, 3540-3544.
24. A. D. Becke, *J. Chem. Phys.*, 1988, **88**, 2547-2553.
25. C. Lee, W. Yang and R. G. Parr, *Phys. Rev. B*, 1988, **37**, 785-789.
26. B. G. Levine, J. D. Coe and T. J. Martínez, *J. Phys. Chem. B*, 2008, **112**, 405-413.
27. R. Crespo-Otero and M. Barbatti, *Theor. Chem. Acc.*, 2012, **131**, 1237.
28. M. Barbatti, *WIREs: Comp. Mol. Sci.*, 2011, **1**, 620-633.
29. J. C. Tully, *J. Chem. Phys.*, 1990, **93**, 1061-1071.
30. G. Granucci and M. Persico, *J. Chem. Phys.*, 2007, **126**, 134114-134111.
31. F. Plasser, R. Crespo-Otero, M. Pederzoli, J. Pittner, H. Lischka and M. Barbatti, *J. Chem. Theory Comput.*, 2014, **10**, 1395-1405.
32. S. Hammes-Schiffer and J. C. Tully, *J. Chem. Phys.*, 1994, **101**, 4657-4667.
33. J. Zheng, R. Meana-Pañeda and D. G. Truhlar, *J. Phys. Chem. Lett.*, 2014, **5**, 2039-2043.
34. R. Ahlrichs, M. Bär, M. Häser, H. Horn and C. Kölmel, *Chem. Phys. Lett.*, 1989, **162**, 165-169.
35. M. Barbatti, G. Granucci, M. Ruckenbauer, F. Plasser, R. Crespo-Otero, J. Pittner, M. Persico and H. Lischka, *NEWTON-X: a package for Newtonian dynamics close to the crossing seam*, 2013, www.newtonx.org.
36. M. Barbatti, M. Ruckenbauer, F. Plasser, J. Pittner, G. Granucci, M. Persico and H. Lischka, *WIREs: Comp. Mol. Sci.*, 2014, **4**, 26-33.
37. M. J. Frisch, G. W. Trucks, H. B. Schlegel, G. E. Scuseria, M. A. Robb, J. R. Cheeseman, G. Scalmani, V. Barone, B. Mennucci, G. A. Petersson, H. Nakatsuji, M. Caricato, X. Li, H. P. Hratchian, A. F. Izmaylov, J. Bloino, G. Zheng, J. L. Sonnenberg, M. Hada, M. Ehara, K. Toyota, R. Fukuda, J. Hasegawa, M. Ishida, T. Nakajima, Y. Honda, O. Kitao, H. Nakai, T. Vreven, J. Montgomery, J. A., J. E. Peralta, F. Ogliaro, M. Bearpark, J. J. Heyd, E. Brothers, K. N. Kudin, V. N. Staroverov, R. Kobayashi, J. Normand, K. Raghavachari, A. Rendell, J. C. Burant, S. S. Iyengar, J. Tomasi, M. Cossi, N. Rega, N. J. Millam, M. Klene, J. E. Knox, J. B. Cross, V. Bakken, C. Adamo, J. Jaramillo, R. Gomperts, R. E. Stratmann, O. Yazyev, A. J. Austin, R. Cammi, C. Pomelli, J. W. Ochterski, R. L. Martin, K. Morokuma, V. G. Zakrzewski, G. A. Voth, P. Salvador, J. J. Dannenberg, S. Dapprich, A. D. Daniels, Ö. Farkas, J. B. Foresman, J. V. Ortiz, J. Cioslowski and D. J. Fox, *Gaussian 09, Revision D.01. Gaussian, Inc., Wallingford CT*, 2013.

38. L. Serrano-Andrés and M. Merchán, *Chem. Phys. Lett.*, 2006, **418**, 569-575.
39. K. Ando, S. Hayashi and S. Kato, *Phys. Chem. Chem. Phys.*, 2011, **13**, 11118-11127.
40. X.-f. Yu, S. Yamazaki and T. Taketsugu, *J. Chem. Theory Comput.*, 2011, **7**, 1006-1015.
41. M. Moreno, A. Douhal, J. M. Lluch, O. Castaño and L. M. Frutos, *J. Phys. Chem. A*, 2001, **105**, 3887-3893.
42. Y. Tawada, T. Tsuneda, S. Yanagisawa, T. Yanai and K. Hirao, *J. Chem. Phys.*, 2004, **120**, 8425-8433.
43. M. S. Gordon and M. W. Schmidt, in *Theory and Applications of Computational Chemistry the first forty years*, eds. C. E. Dykstra, G. Frenking, K. S. Kim and G. E. Scuseria, Elsevier, Amsterdam, 2005, pp. 1167-1189.
44. T. Minami, M. Nakano and F. Castet, *J. Phys. Chem. Lett.*, 2011, **2**, 1725-1730.
45. C. L. Janssen and I. M. B. Nielsen, *Chem. Phys. Lett.*, 1998, **290**, 423-430.
46. I. M. B. Nielsen and C. L. Janssen, *Chem. Phys. Lett.*, 1999, **310**, 568-576.
47. C. Hättig, A. Köhn and K. Hald, *J. Chem. Phys.*, 2002, **116**, 5401-5410.

SECTION S13. CARTESIAN COORDINATES

Cartesian coordinates of all species reported in this work in Å.

ADC(2)/SV(P)

```
   30
Min S0
 N   -0.072922    0.000000   -0.016406
 C    0.028306   -0.000000    1.320277
 C    1.246744    0.000000    2.072563
 C    2.446810   -0.000000    1.336879
 C    2.351535    0.000000   -0.053933
 C    1.087102   -0.000000   -0.683022
 C    0.873023   -0.000000    3.449865
 C   -0.514665   -0.000000    3.474512
 N   -1.017713    0.000000    2.199948
 N   -3.762906   -0.000000    1.199494
 C   -4.922764    0.000000    1.866384
 C   -6.187330   -0.000000    1.237577
 C   -6.282899    0.000000   -0.153217
 C   -5.082994   -0.000000   -0.889172
 C   -3.864396    0.000000   -0.137159
 C   -4.709566    0.000000   -2.266554
 C   -3.321882    0.000000   -2.291507
 N   -2.818550   -0.000000   -1.017046
 H   -2.023749   -0.000000    1.924197
 H   -1.812480    0.000000   -0.741479
 H    3.423180    0.000000    1.837266
 H    3.254030   -0.000000   -0.675078
 H    1.017991    0.000000   -1.779252
 H   -7.259382   -0.000000   -0.653382
 H   -7.089685    0.000000    1.858924
 H   -4.853380   -0.000000    2.962598
 H    1.531898    0.000000    4.320080
 H   -1.189307    0.000000    4.334190
 H   -2.647444   -0.000000   -3.151343
 H   -5.368636   -0.000000   -3.136623
   30
Min S1-N
 N   -0.167912    0.032907   -0.048484
 C    0.002014    0.008862    1.282896
 C    1.178288   -0.012671    2.033416
 C    2.437192   -0.011755    1.343128
 C    2.303354    0.016362   -0.075475
 C    1.066923    0.036839   -0.710018
 C    0.763732   -0.032070    3.417379
 C   -0.670199   -0.020215    3.460122
 N   -1.098298    0.004261    2.197744
 N   -3.676365    0.026028    1.205578
 C   -4.827696    0.032037    1.891674
 C   -6.096953    0.017002    1.279458
 C   -6.209336   -0.006514   -0.111820
 C   -5.022140   -0.012681   -0.865521
 C   -3.787674    0.005481   -0.133551
 C   -4.665652   -0.032667   -2.245709
 C   -3.276097   -0.025370   -2.283534
 N   -2.753765   -0.002757   -1.019209
 H   -2.130200    0.017933    1.864024
 H   -1.719682    0.011580   -0.736662
 H    3.404702   -0.028303    1.855758
 H    3.202149    0.022139   -0.705838
 H    1.007018    0.059481   -1.806422
 H   -7.193444   -0.019285   -0.596553
 H   -6.991161    0.023610    1.912303
 H   -4.742659    0.049889    2.986795
 H    1.412918   -0.052385    4.297782
 H   -1.347442   -0.028871    4.316479
 H   -2.611709   -0.035189   -3.151381
 H   -5.333845   -0.049632   -3.108657
   30
Min S1-D
 N   -0.093486    0.040007    0.024433
 C    0.019255    0.009187    1.384843
 C    1.217330   -0.013210    2.060566
 C    2.467834   -0.011647    1.340616
 C    2.332129    0.017015   -0.067611
 C    1.095267    0.039060   -0.704114
 C    0.824464   -0.035856    3.468637
 C   -0.598057   -0.021370    3.504463
 N   -1.098256    0.006917    2.263494
 N   -3.747716    0.024574    1.129864
 C   -4.871917    0.032149    1.872534
 C   -6.130341    0.018096    1.261541
 C   -6.247006   -0.005982   -0.135409
 C   -5.078940   -0.013673   -0.911524
 C   -3.799095    0.004215   -0.225609
 C   -4.730478   -0.035380   -2.283533
 C   -3.329539   -0.028395   -2.303755
 N   -2.755335   -0.004500   -1.059070
 H   -2.801519    0.028113    1.594298
 H   -1.034093    0.027744   -0.441856
 H    3.441726   -0.029779    1.841708
 H    3.226112    0.021032   -0.703241
 H    0.985356    0.061175   -1.793254
 H   -7.238653   -0.018825   -0.605267
 H   -7.020979    0.025188    1.897822
 H   -4.735379    0.048989    2.958592
 H    1.495366   -0.060149    4.332997
 H   -1.245329   -0.031107    4.386305
 H   -2.689633   -0.039983   -3.193090
 H   -5.400678   -0.053607   -3.145169
   30
Min S1-S
 N    0.049040    0.006402    0.058329
 C    0.172676   -0.015025    1.373956
 C    1.404519   -0.019214    2.085462
 C    2.601403   -0.003245    1.354553
 C    2.481378    0.017843   -0.039454
 C    1.207490    0.022852   -0.636869
 C    1.030597   -0.037815    3.463199
 C   -0.409999   -0.045857    3.476053
 N   -0.926696   -0.034630    2.265621
 N   -3.901148    0.116408    1.184999
 C   -5.112876    0.054395    1.881911
 C   -6.323386    0.013898    1.197107
 C   -6.411382   -0.016584   -0.207695
 C   -5.152476   -0.008144   -0.925937
 C   -3.959686    0.028601   -0.190138
 C   -4.756840   -0.042162   -2.295564
 C   -3.366284   -0.017172   -2.338334
 N   -2.890897    0.033161   -1.040414
 H   -3.001626    0.017511    1.663213
 H   -1.908471    0.039213   -0.751850
 H    3.582786   -0.006892    1.843900
 H    3.370743    0.031502   -0.677005
 H    1.112078    0.040881   -1.728602
 H   -7.376950   -0.051923   -0.727617
 H   -7.239640   -0.002771    1.803244
 H   -5.038486    0.092448    2.974409
 H    1.689779   -0.043761    4.336950
 H   -1.044426   -0.059891    4.368715
 H   -2.680152   -0.037934   -3.187089
 H   -5.414961   -0.080623   -3.167668
   30
TS S1-N
 N   -0.235970    0.046128   -0.074138
 C   -0.083797    0.002127    1.268079
 C    1.096087   -0.036043    2.002684
 C    2.355743   -0.034151    1.314095
 C    2.226908    0.015572   -0.103681
 C    0.992747    0.051990   -0.741738
 C    0.678782   -0.074300    3.393386
```

```
 C   -0.748484   -0.053603    3.424576
 N   -1.196126   -0.007380    2.165363
 N   -3.606199    0.022582    1.208326
 C   -4.740572    0.024955    1.925487
 C   -6.011128    0.002627    1.324751
 C   -6.137618   -0.025861   -0.067413
 C   -4.964158   -0.028155   -0.840691
 C   -3.711715   -0.000623   -0.134181
 C   -4.621359   -0.051670   -2.221571
 C   -3.228930   -0.036573   -2.267919
 N   -2.682891   -0.005986   -1.013583
 H   -2.386154    0.016285    1.750424
 H   -1.573047    0.019165   -0.688041
 H    3.322452   -0.061888    1.828452
 H    3.127856    0.024984   -0.730886
 H    0.930407    0.090754   -1.837510
 H   -7.128799   -0.046059   -0.537360
 H   -6.899264    0.006142    1.965522
 H   -4.626240    0.044795    3.016720
 H    1.330483   -0.113426    4.271805
 H   -1.419596   -0.071473    4.286790
 H   -2.578533   -0.046050   -3.146920
 H   -5.297856   -0.076436   -3.077887
   30
TS S1-D
 N   -0.085678    0.015968   -0.082933
 C   -0.045134    0.003147    1.280069
 C    1.110788   -0.020709    2.042719
 C    2.396981   -0.037059    1.385888
 C    2.341841   -0.026815   -0.028011
 C    1.147043   -0.001858   -0.740396
 C    0.646274   -0.022636    3.414934
 C   -0.769618   -0.000400    3.389026
 N   -1.180741    0.015943    2.105166
 N   -3.594190    0.003872    1.137754
 C   -4.700478   -0.027474    1.904693
 C   -5.996083   -0.053165    1.363261
 C   -6.182254   -0.048144   -0.025081
 C   -5.041056   -0.016018   -0.841384
 C   -3.749836    0.009061   -0.197586
 C   -4.749153    0.000249   -2.232072
 C   -3.344582    0.032259   -2.314665
 N   -2.732941    0.038133   -1.103506
 H   -2.303272    0.018703    1.685395
 H   -1.012401    0.029958   -0.586424
 H    3.342871   -0.057716    1.938490
 H    3.272833   -0.039010   -0.608696
 H    1.102110    0.006502   -1.834458
 H   -7.192902   -0.069365   -0.452731
 H   -6.855459   -0.077812    2.041952
 H   -4.540674   -0.032227    2.990938
 H    1.266263   -0.037427    4.316325
 H   -1.478918    0.004702    4.220309
 H   -2.745990    0.052100   -3.232331
 H   -5.455412   -0.009134   -3.066114
   30
X10
 N    0.252330   -0.235250    0.093433
 C    0.399265   -0.023746    1.381457
 C    1.622218    0.216116    2.048215
 C    2.802768    0.270048    1.300388
 C    2.667125    0.048225   -0.077184
 C    1.398871   -0.204032   -0.626784
 C    1.262853    0.351268    3.429281
 C   -0.185290    0.190555    3.480421
 N   -0.699517   -0.017075    2.305258
 N   -4.249739   -0.551657    1.233688
 C   -5.548831   -0.389857    1.811309
 C   -6.674293   -0.146900    1.037237
 C   -6.633620    0.086509   -0.351042
 C   -5.292794    0.073152   -0.934944
 C   -4.197240   -0.166642   -0.120899
 C   -4.773373    0.262828   -2.249919
 C   -3.392102    0.115082   -2.189824
 N   -3.048555   -0.164308   -0.883359
 H   -3.482635   -0.200718    1.804133
 H   -2.105486   -0.307171   -0.537911
 H    3.778926    0.474652    1.755421
 H    3.539347    0.068519   -0.735819
 H    1.291907   -0.390732   -1.701037
 H   -7.528836    0.311899   -0.942105
 H   -7.639775   -0.126123    1.564140
 H   -5.604938   -0.616455    2.883428
 H    1.926763    0.541903    4.278352
 H   -0.797680    0.243719    4.385861
 H   -2.632224    0.213557   -2.965829
 H   -5.346715    0.482744   -3.155145
```

ADC(2)/TZVP

```
   30
Min S0
 N   -0.093929    0.046863   -0.010231
 C    0.021030    0.051031    1.324198
 C    1.232977   -0.042222    2.068951
 C    2.424477    0.012320    1.335580
 C    2.327685   -0.010568   -0.047550
 C    1.070294    0.022987   -0.671186
 C    0.866691   -0.010442    3.443489
 C   -0.511529    0.017166    3.477084
 N   -1.022073    0.082548    2.206159
 N   -3.742400   -0.046891    1.193618
 C   -4.906606   -0.022963    1.854532
 C   -6.163899    0.010714    1.230712
 C   -6.260649   -0.012253   -0.152445
 C   -5.069082    0.042166   -0.885723
 C   -3.857208   -0.051023   -0.140842
 C   -4.702708    0.010579   -2.260242
 C   -3.324472   -0.017488   -2.293664
 N   -2.813957   -0.082465   -1.022710
 H   -2.017714    0.030729    1.936128
 H   -1.818259   -0.030676   -0.752641
 H    3.390397   -0.012025    1.826440
 H    3.215846    0.005637   -0.665722
 H    1.000616    0.008550   -1.753320
 H   -7.226573    0.012176   -0.643304
 H   -7.052041   -0.005528    1.848861
 H   -4.836894   -0.008775    2.936729
 H    1.519648   -0.062378    4.299232
 H   -1.174235    0.039071    4.327822
 H   -2.661758   -0.039023   -3.144391
 H   -5.355689    0.062180   -3.115989
   30
Min S1-N
 N   -0.173823    0.024340   -0.056565
 C    0.002940   -0.015430    1.275743
 C    1.167561    0.041497    2.025310
 C    2.424562   -0.055442    1.347285
 C    2.299629    0.015168   -0.064472
 C    1.073402    0.030792   -0.702304
 C    0.753498   -0.043526    3.400758
 C   -0.672164   -0.008018    3.445280
 N   -1.104225    0.008294    2.187388
 N   -3.665299    0.022628    1.207965
 C   -4.822816    0.025157    1.886375
 C   -6.083771    0.008749    1.276176
 C   -6.194418    0.021072   -0.108103
 C   -5.014931   -0.057021   -0.855715
 C   -3.786820    0.030321   -0.129648
 C   -4.663741   -0.039611   -2.232378
 C   -3.283199   -0.030693   -2.276907
 N   -2.754119    0.024744   -1.015709
 H   -2.128030    0.022953    1.862119
 H   -1.730112    0.016407   -0.744195
 H    3.374902    0.007323    1.859551
 H    3.187981    0.011296   -0.686029
 H    1.023322    0.080396   -1.784134
 H   -7.166913    0.008214   -0.586395
 H   -6.965318    0.045042    1.902690
 H   -4.740117    0.015840    2.967779
 H    1.394900   -0.079015    4.269816
 H   -1.341520   -0.024078    4.289690
 H   -2.629256   -0.021674   -3.134902
 H   -5.326030   -0.091680   -3.080771
   30
```

```
Min S1-D
 N   -0.105163    0.344305    0.037242
 C    0.010025    0.099873    1.377937
 C    1.198182   -0.013625    2.041366
 C    2.433408   -0.120120    1.311613
 C    2.301703    0.048997   -0.078119
 C    1.076440    0.215160   -0.699728
 C    0.807156   -0.184650    3.433592
 C   -0.599416   -0.046321    3.489393
 N   -1.110175    0.130257    2.265081
 N   -3.746593    0.068514    1.148298
 C   -4.881308    0.117801    1.871476
 C   -6.122943    0.058270    1.248296
 C   -6.220444   -0.008613   -0.142084
 C   -5.050172   -0.119441   -0.893460
 C   -3.785295    0.022321   -0.204957
 C   -4.689854   -0.178118   -2.256092
 C   -3.297319   -0.155339   -2.266438
 N   -2.728892   -0.033059   -1.020946
 H   -2.815833    0.121834    1.616805
 H   -1.028198    0.208592   -0.424804
 H    3.394346   -0.206112    1.800167
 H    3.174126   -0.011947   -0.717648
 H    0.972266    0.333035   -1.769075
 H   -7.194683   -0.058848   -0.615044
 H   -7.008896    0.122658    1.864525
 H   -4.759876    0.169462    2.944265
 H    1.468876   -0.349296    4.272990
 H   -1.233859   -0.104343    4.361133
 H   -2.656884   -0.191809   -3.137154
 H   -5.342316   -0.279438   -3.108420
   30
Min S1-S
 N    0.035375    0.298707    0.055577
 C    0.153971    0.035106    1.343138
 C    1.367229   -0.199570    2.033784
 C    2.566731    0.003482    1.349302
 C    2.467063    0.267213   -0.013437
 C    1.209365    0.416268   -0.606524
 C    0.980299   -0.411502    3.384975
 C   -0.453055   -0.394815    3.387949
 N   -0.963402   -0.130005    2.204066
 N   -3.877418    0.500867    1.148235
 C   -5.094261    0.445651    1.850886
 C   -6.292020    0.241267    1.189916
 C   -6.370927   -0.074107   -0.172132
 C   -5.120284   -0.086402   -0.889899
 C   -3.939745    0.082596   -0.171171
 C   -4.721195   -0.404118   -2.217141
 C   -3.341223   -0.334317   -2.272343
 N   -2.866042   -0.052702   -1.004906
 H   -3.018906    0.286272    1.650472
 H   -1.901419    0.133758   -0.749053
 H    3.530489   -0.114835    1.830247
 H    3.352123    0.409785   -0.618221
 H    1.129868    0.627526   -1.665282
 H   -7.321667   -0.202848   -0.674471
 H   -7.192958    0.269321    1.793999
 H   -5.035084    0.699604    2.900534
 H    1.620755   -0.606320    4.234734
 H   -1.089455   -0.553589    4.248035
 H   -2.659800   -0.537221   -3.081489
 H   -5.368285   -0.623598   -3.052398
   30
TS S1-N
 N   -0.245693    0.308283   -0.065501
 C   -0.091329    0.069480    1.258535
 C    1.076996   -0.054184    1.985091
 C    2.324011   -0.158452    1.291870
 C    2.209813    0.082562   -0.101128
 C    0.991370    0.269786   -0.726168
 C    0.653973   -0.256616    3.354089
 C   -0.757011   -0.117308    3.401755
 N   -1.207788    0.080347    2.159073
 N   -3.595936    0.130472    1.216088
 C   -4.737968    0.165976    1.920472
 C   -5.995333    0.074339    1.318430
 C   -6.111838   -0.029586   -0.064062
 C   -4.939814   -0.137324   -0.818258
 C   -3.701056    0.038046   -0.121765
 C   -4.594064   -0.227458   -2.191191
 C   -3.211102   -0.191614   -2.239504
 N   -2.668055   -0.033850   -0.992245
 H   -2.397999    0.145047    1.751513
 H   -1.591526    0.103333   -0.691222
 H    3.275761   -0.233642    1.800153
 H    3.096656    0.075685   -0.725177
 H    0.944693    0.453845   -1.793606
 H   -7.087920   -0.108143   -0.528465
 H   -6.875156    0.137561    1.944481
 H   -4.631060    0.245211    2.995707
 H    1.293132   -0.438304    4.207169
 H   -1.420731   -0.195053    4.248564
 H   -2.564237   -0.238514   -3.102209
 H   -5.257762   -0.361494   -3.029549
   30
TS S1-D
 N   -0.096073    0.390878   -0.068778
 C   -0.091693    0.102799    1.267778
 C    1.028998   -0.192184    2.008706
 C    2.310107   -0.270469    1.359225
 C    2.265624   -0.160077   -0.043342
 C    1.104290    0.120901   -0.739201
 C    0.580831   -0.176168    3.380798
 C   -0.792379    0.122150    3.379029
 N   -1.198971    0.329214    2.109394
 N   -3.571438    0.289139    1.141552
 C   -4.664779    0.102588    1.908372
 C   -5.922614   -0.174655    1.373330
 C   -6.095780   -0.270436   -0.007587
 C   -4.962086   -0.179699   -0.815701
 C   -3.722599    0.190215   -0.189404
 C   -4.675337   -0.177112   -2.203278
 C   -3.308202    0.097858   -2.298642
 N   -2.717013    0.340437   -1.096731
 H   -2.319680    0.392637    1.690927
 H   -1.011022    0.385891   -0.574776
 H    3.214407   -0.541344    1.887573
 H    3.170043   -0.294460   -0.625141
 H    1.072140    0.233189   -1.813784
 H   -7.072249   -0.493017   -0.423245
 H   -6.766031   -0.265436    2.044930
 H   -4.511960    0.171656    2.979331
 H    1.182253   -0.367236    4.258100
 H   -1.483512    0.168092    4.205946
 H   -2.725127    0.170274   -3.206811
 H   -5.349894   -0.391995   -3.018042
   30
X10
 N    0.244182   -0.221822    0.096144
 C    0.404022   -0.007240    1.381134
 C    1.628688    0.159274    2.054493
 C    2.792484    0.276121    1.299423
 C    2.661962    0.037809   -0.067225
 C    1.398425   -0.193525   -0.615063
 C    1.267414    0.366266    3.418718
 C   -0.168433    0.193433    3.477575
 N   -0.696063   -0.016550    2.307043
 N   -4.252936   -0.565846    1.225458
 C   -5.550795   -0.389611    1.802850
 C   -6.667413   -0.149720    1.030108
 C   -6.618164    0.134436   -0.341308
 C   -5.293497    0.048237   -0.937357
 C   -4.201106   -0.156965   -0.121872
 C   -4.773845    0.266400   -2.243034
 C   -3.401807    0.105063   -2.188840
 N   -3.052595   -0.166085   -0.882441
 H   -3.499932   -0.197724    1.791965
 H   -2.115387   -0.306228   -0.541194
 H    3.758911    0.464287    1.750775
 H    3.521085    0.074549   -0.722039
 H    1.296768   -0.386301   -1.674516
 H   -7.508913    0.283904   -0.936011
 H   -7.617792   -0.121194    1.555328
```

```
 H   -5.608143   -0.620773    2.857989
 H    1.925067    0.555429    4.257804
 H   -0.773088    0.243478    4.371335
 H   -2.650237    0.222527   -2.949526
 H   -5.340104    0.478480   -3.137485
```

CC2/TZVP

```
   30
Min S0
 N   -0.097933    0.047204   -0.013832
 C    0.019661    0.050446    1.323434
 C    1.234281   -0.044093    2.070068
 C    2.425038    0.011485    1.336487
 C    2.330231   -0.010777   -0.052238
 C    1.073211    0.022816   -0.675281
 C    0.866418   -0.010661    3.447688
 C   -0.511965    0.017969    3.482494
 N   -1.025915    0.084235    2.205891
 N   -3.738290   -0.047235    1.197196
 C   -4.909477   -0.022845    1.858568
 C   -6.166459    0.010855    1.235452
 C   -6.261183   -0.011394   -0.153277
 C   -5.070382    0.044106   -0.886786
 C   -3.855804   -0.050409   -0.140080
 C   -4.702441    0.010599   -2.264384
 C   -3.324054   -0.018011   -2.299108
 N   -2.810186   -0.084238   -1.022477
 H   -2.024801    0.030568    1.933622
 H   -1.811308   -0.030548   -0.750151
 H    3.392123   -0.011326    1.826953
 H    3.220094    0.006363   -0.669056
 H    1.001940    0.009268   -1.758093
 H   -7.228235    0.011462   -0.643803
 H   -7.056363   -0.006247    1.852210
 H   -4.838283   -0.009414    2.941385
 H    1.521702   -0.062854    4.302678
 H   -1.176474    0.039751    4.332625
 H   -2.659489   -0.039792   -3.149192
 H   -5.357672    0.062716   -3.119416
   30
Min S1-N
 N   -0.175497    0.025974   -0.060930
 C    0.002200   -0.017696    1.274901
 C    1.168840    0.040357    2.024515
 C    2.424619   -0.054121    1.347758
 C    2.301733    0.015103   -0.067299
 C    1.076928    0.030154   -0.708212
 C    0.754895   -0.044621    3.404924
 C   -0.674217   -0.008158    3.448764
 N   -1.110917    0.009137    2.189441
 N   -3.661232    0.022469    1.211120
 C   -4.825089    0.025850    1.890705
 C   -6.086038    0.008865    1.281524
 C   -6.194594    0.020429   -0.108190
 C   -5.016027   -0.058493   -0.856588
 C   -3.784791    0.030150   -0.129450
 C   -4.663480   -0.039949   -2.236622
 C   -3.283117   -0.030823   -2.283002
 N   -2.749811    0.026718   -1.016041
 H   -2.141714    0.024008    1.860735
 H   -1.720989    0.015934   -0.741624
 H    3.375014    0.008632    1.860196
 H    3.192329    0.010660   -0.687120
 H    1.025879    0.080537   -1.790208
 H   -7.168181    0.007829   -0.585828
 H   -6.969304    0.045243    1.906926
 H   -4.740328    0.016530    2.972517
 H    1.398526   -0.079642    4.274013
 H   -1.343590   -0.024085    4.294282
 H   -2.628020   -0.021759   -3.141023
 H   -5.327951   -0.091186   -3.084486
   30
Min S1-D
 N   -0.106912    0.349093    0.035130
 C    0.010161    0.100111    1.377125
 C    1.201470   -0.014167    2.040976
 C    2.433351   -0.119041    1.313256
 C    2.302854    0.048398   -0.081837
 C    1.079238    0.215280   -0.705289
 C    0.808518   -0.186138    3.439011
 C   -0.600150   -0.046574    3.494164
 N   -1.115063    0.131142    2.265671
 N   -3.743574    0.067934    1.151421
 C   -4.884917    0.118553    1.875980
 C   -6.125888    0.058682    1.253511
 C   -6.221630   -0.008951   -0.143372
 C   -5.052019   -0.120662   -0.894068
 C   -3.784923    0.021117   -0.205113
 C   -4.688628   -0.177751   -2.261640
 C   -3.297065   -0.155593   -2.273600
 N   -2.724343   -0.033258   -1.021158
 H   -2.810116    0.122816    1.620586
 H   -1.033115    0.206468   -0.426113
 H    3.394989   -0.205870    1.801094
 H    3.177889   -0.013021   -0.718901
 H    0.973225    0.334084   -1.774642
 H   -7.197157   -0.058367   -0.615727
 H   -7.014160    0.123605    1.867618
 H   -4.761282    0.169555    2.949384
 H    1.472414   -0.350657    4.278138
 H   -1.237249   -0.104541    4.364693
 H   -2.653831   -0.192164   -3.143063
 H   -5.343659   -0.280086   -3.113028
   30
Min S1-S
 N    0.028932    0.298597    0.053472
 C    0.149552    0.032496    1.341642
 C    1.367212   -0.201602    2.034959
 C    2.565573    0.002654    1.350461
 C    2.466466    0.268711   -0.018086
 C    1.210478    0.417187   -0.610730
 C    0.980821   -0.412521    3.388441
 C   -0.455123   -0.395880    3.392162
 N   -0.972823   -0.132253    2.205261
 N   -3.872246    0.513509    1.148389
 C   -5.094500    0.448852    1.854163
 C   -6.291488    0.240506    1.193770
 C   -6.369962   -0.073212   -0.173390
 C   -5.122428   -0.084084   -0.891149
 C   -3.939457    0.085377   -0.171408
 C   -4.720263   -0.406133   -2.221140
 C   -3.339726   -0.336275   -2.276806
 N   -2.862393   -0.054964   -1.004791
 H   -3.016554    0.276984    1.650997
 H   -1.897858    0.141887   -0.749163
 H    3.530690   -0.113795    1.831252
 H    3.353763    0.410724   -0.621152
 H    1.128756    0.629441   -1.670111
 H   -7.322255   -0.201732   -0.673845
 H   -7.193578    0.263084    1.797286
 H   -5.033380    0.702299    2.904359
 H    1.622998   -0.606539    4.238854
 H   -1.090726   -0.554329    4.254189
 H   -2.656016   -0.542553   -3.083998
 H   -5.368342   -0.624964   -3.056508
   30
TS S1-N
 N   -0.246666    0.346180   -0.062519
 C   -0.089157    0.085106    1.259864
 C    1.081959   -0.060457    1.981607
 C    2.324045   -0.166828    1.284371
 C    2.209433    0.088579   -0.109237
 C    0.992622    0.296814   -0.730927
 C    0.660756   -0.281457    3.353322
 C   -0.752802   -0.129028    3.407077
 N   -1.207957    0.094264    2.167609
 N   -3.600926    0.147156    1.223107
 C   -4.753338    0.186715    1.921903
 C   -6.008071    0.083605    1.316848
 C   -6.116587   -0.037086   -0.070468
 C   -4.941582   -0.149899   -0.818415
 C   -3.703896    0.037098   -0.116915
 C   -4.587738   -0.249501   -2.192997
 C   -3.205384   -0.208804   -2.237537
 N   -2.663792   -0.039163   -0.982919
```

```
 H   -2.389122    0.163670    1.766902
 H   -1.584296    0.119014   -0.679842
 H    3.276671   -0.257388    1.788957
 H    3.096359    0.076813   -0.734247
 H    0.942839    0.492815   -1.796398
 H   -7.091543   -0.122644   -0.537932
 H   -6.892485    0.152146    1.936970
 H   -4.650878    0.279739    2.997292
 H    1.302214   -0.483472    4.201564
 H   -1.415572   -0.211822    4.255063
 H   -2.552981   -0.259495   -3.096629
 H   -5.249098   -0.394238   -3.032535
   30
TS S1-D
 N   -0.096583    0.438142   -0.065024
 C   -0.094497    0.134737    1.271035
 C    1.022602   -0.196681    2.007024
 C    2.295166   -0.306148    1.353254
 C    2.249874   -0.190441   -0.053261
 C    1.095334    0.126699   -0.744526
 C    0.575221   -0.188229    3.385250
 C   -0.791111    0.145134    3.389657
 N   -1.197646    0.383259    2.119182
 N   -3.569410    0.322804    1.143504
 C   -4.665313    0.109709    1.912106
 C   -5.914459   -0.199010    1.378151
 C   -6.081769   -0.308966   -0.009451
 C   -4.950746   -0.196810   -0.815519
 C   -3.718644    0.208218   -0.189065
 C   -4.661317   -0.191866   -2.208034
 C   -3.302010    0.117058   -2.304777
 N   -2.713154    0.381792   -1.098011
 H   -2.316581    0.446061    1.699880
 H   -1.016184    0.436784   -0.568821
 H    3.194583   -0.603970    1.876008
 H    3.149144   -0.352367   -0.637214
 H    1.060068    0.243855   -1.818889
 H   -7.053488   -0.556772   -0.424233
 H   -6.758662   -0.306568    2.047463
 H   -4.513655    0.188853    2.983301
 H    1.175334   -0.405562    4.258510
 H   -1.481188    0.199100    4.217774
 H   -2.718675    0.200700   -3.212579
 H   -5.331985   -0.425890   -3.022166
   30
X10
 N    0.245819   -0.222681    0.097028
 C    0.407298   -0.014446    1.382624
 C    1.632849    0.159107    2.056603
 C    2.798315    0.272136    1.303510
 C    2.668802    0.042252   -0.073278
 C    1.406756   -0.191488   -0.618628
 C    1.269598    0.380287    3.422966
 C   -0.170305    0.202609    3.486616
 N   -0.700568   -0.046301    2.320009
 N   -4.244692   -0.584299    1.228377
 C   -5.553228   -0.398139    1.804771
 C   -6.667305   -0.145354    1.032411
 C   -6.623509    0.135155   -0.342685
 C   -5.298540    0.052792   -0.936520
 C   -4.207229   -0.166694   -0.124321
 C   -4.775301    0.275423   -2.247078
 C   -3.403572    0.104273   -2.195086
 N   -3.052430   -0.169445   -0.885611
 H   -3.514597   -0.148303    1.782466
 H   -2.118234   -0.322624   -0.543671
 H    3.767660    0.455239    1.753000
 H    3.529835    0.074343   -0.729948
 H    1.296866   -0.379473   -1.680589
 H   -7.517564    0.271878   -0.937529
 H   -7.616132   -0.109922    1.559932
 H   -5.618703   -0.636286    2.858657
 H    1.933844    0.561112    4.260313
 H   -0.773455    0.247333    4.382209
 H   -2.650375    0.229053   -2.953173
 H   -5.343161    0.482591   -3.143175
```
